# Disease progression in bipolar disorder in relation to white matter microstructure: A comprehensive approach based on staging models

**DOI:** 10.1192/j.eurpsy.2025.10105

**Published:** 2025-09-15

**Authors:** Katharina Thiel, Kira Flinkenflügel, Dominik Grotegerd, Christoph Jurischka, Julia Hubbert, Tim Hahn, Elisabeth J. Leehr, Hannah Meinert, Elisabeth Schrammen, Florian Thomas-Odenthal, Paula Usemann, Lea Teutenberg, Benjamin Straube, Nina Alexander, Hamidreza Jamalabadi, Andreas Jansen, Frederike Stein, Michael Bauer, Andrea Pfennig, Eva Mennigen, Philipp Kanske, Katharina Förster, Igor Nenadić, Tilo Kircher, Susanne Meinert, Udo Dannlowski

**Affiliations:** 1 Institute for Translational Psychiatry, University of Münster, Münster, German; 2 Department of Clinical Psychology and Psychotherapy, University of Göttingen, Göttingen, Germany; 3 Department of Psychiatry and Psychotherapy, University of Marburg, Marburg, Germany; 4 Center for Mind, Brain and Behavior (CMBB), University of Marburg, Marburg, Germany; 5 Core-Facility Brainimaging, Faculty of Medicine, University of Marburg, Marburg, Germany; 6 Department of Psychiatry and Psychotherapy, Faculty of Medicine, TUD Dresden University of Technology, Dresden, Germany; 7 Clinical Psychology and Behavioral Neuroscience, Faculty of Psychology, TUD Dresden University of Technology, Dresden, Germany.; 8 Department of Psychology, Faculty of Psychology and Educational Sciences, Babeș-Bolyai University, Cluj-Napoca, Romania; 9 Institute of Translational Neuroscience, University of Münster, Münster, Germany; 10 Department of Psychiatry, Medical School and University Medical Center OWL, Protestant Hospital of the Bethel Foundation, Bielefeld University

**Keywords:** bipolar disorder, diffusion tensor imaging, neuroprogression, staging, white matter microstructure

## Abstract

**Background:**

Bipolar disorder (BD) is assumed to follow a progressive course, conceptualized through staging models. It is unclear whether white matter (WM) microstructure abnormalities, central to BD pathophysiology, parallel this development throughout disease progression. This study explored the link between WM and disease progression in BD, using a comprehensive approach based on clinical staging models.

**Methods:**

This cross-sectional diffusion tensor-imaging study included 153 BD patients and 153 healthy controls (HCs) matched for age, sex, and study site. Using tract-based spatial statistics (TBSS), we examined associations between WM integrity and three criteria: (1) number of manic episodes, (2) remission quality between episodes, and (3) inter-episode global functioning.

**Results:**

Analyses revealed significant fractional anisotropy (FA) differences between early and late stages of BD based on the number of manic episodes (*p*_tfce-FWE_ = 0.003), but not on remission quality (*p*_tfce-FWE_ = 0.075). However, compared to HC, BD patients with persistent symptoms between episodes showed more widespread FA differences (*p*_tfce-FWE_ < 0.001) than those with stable remission (*p*_tfce-FWE_ = 0.031). Regression analyses indicated a positive association between global functioning and FA in euthymic BD patients (*p_tfce-FWE_* < 0.001).

**Conclusions:**

Results indicated more severe WM disruptions in patients at advanced stages compared to earlier stages of the disease. While these findings may imply changes occurring with disease progression, the cross-sectional design cannot rule out that they instead reflect stable clinical subtypes of varying severity. The results highlight the clinical relevance of WM alterations and the need for longitudinal studies to better understand the neurobiology and complexity of BD.

## Introduction

Bipolar disorder (BD) is a chronic, highly relapsing disorder in which episodes of depression and (hypo)mania alternate with euthymic phases. Growing evidence suggests that BD follows a progressive course [1, 2], forming the basis for clinical staging models [3, 4] which divide the course of disease into different phases in order to better predict prognosis and treatment response and to counteract further disease progression by promoting early interventions [5, 6]. These models agree in describing a prodromal phase, initial full episodes of mania or depression, and later stages marked by more frequent, longer, and severe relapses, potentially leading to a persistent stage characterized by limited symptomatic and functional recovery. The two most discussed models focus on either the number of recurrent episodes and quality of remissions [3] or on inter-episode symptoms and functional impairment [4]. The biological basis for these models lies in the concept of neuroprogression, which assumes that pathological brain changes progress alongside worsening clinical features, including cognitive and functional decline [7]. While several studies have attempted to empirically validate staging models based on clinical features [e.g. 8–15], their biological basis in terms of neuroprogression, for example, by linking stages to brain structural changes, remains insufficiently understood, limiting clinical utility [2, 16, 17].

Instead, a current debate concerns the progression of cognitive functions. Although longitudinal studies [18–24], including meta-analyses [25–27], largely suggest cognitive stability in BD, not proving the assumption of neuroprogression [28–30], cross-sectional studies have been interpreted as evidence of a progressive deterioration of cognitive functions [e.g. 2, 31–35] and the lack of longitudinal evidence has been attributed to methodological limitations [36, 37]. However, although cognitive deficits often correlate with structural and functional brain changes, they only provide an indirect indication of neuroprogression. A comprehensive understanding requires direct examination of underlying neurobiological processes. In this context, white matter (WM) microstructural alterations appear particularly promising, as recent evidence increasingly points to their role in both the pathophysiology of BD [e.g. 38–40] and cognitive performance [41, 42]. To date, however, only one study has compared WM integrity across different stages of BD, identifying lower WM integrity in the sagittal striatum and corpus callosum in later stages of BD compared to earlier stages [43]. Building upon these findings, our study aims to investigate in more depth whether the concept of disease progression can be biologically supported by alterations in WM microstructure, assessed through diffusion tensor imaging (DTI).

Most previous studies on neuroprogressive effects on cognition or function have used a simple classification, comparing patients with their first episode to those with multiple episodes [e.g. 43–47]. However, even if the measure of the number of previous manic episodes is convincing due to its simplicity, intuition, and clinical relevance, this classification reflects only part of the proposed staging models [15, 48, 49] and does not capture crucial aspects of disease progression. It fails to consider the variability in the degree of remission between episodes – ranging from clearly separated episodes to persistent forms – as well as differences in inter-episode functioning [4].

To address these gaps, our study uses different criteria to approximate disease progression based on the staging models postulated by Berk et al. [3] and Kapczinski et al. [4]. Given the challenges of operationalizing and validating detailed staging models, we adopted the International Society for Bipolar Disorders’ (ISBD) recommendation [50] to broadly categorize BD into earlier and later stages. This simplified approach follows the call for caution in the application of complex but still incomplete models [51] and allows the exploratory investigation of WM microstructural changes associated with disease progression. As in previous studies, our first approach was to compare patients after their first manic episode with those who had already experienced multiple manic episodes in their lives. Second, the quality of remission was used, which in the staging models is assumed to decrease as BD progresses [16, 52]. Finally, impairment of the patient’s interepisodic psychosocial functioning was used as an indicator of disease progression, including only euthymic patients. Testing the hypothesis of neuroprogression, we expected patients at later stages of the disease to show WM microstructural alterations that differ from patients at earlier stages. Specifically, we expected BD patients with multiple manic episodes to show lower WM microstructural integrity compared to BD patients who experienced only their first manic episode. Additionally, we hypothesized that BD patients not achieving complete remission between episodes show lower WM microstructural integrity compared to BD patients achieving full remission. Furthermore, greater functional impairment in euthymic patients was expected to relate negatively to WM integrity.

## Methods

### Participants

One hundred fifty-three BD patients (*n* = 79 female, *M_age_* = 41.0 years, *SD_age_* = 12.0 years) and 153 HCs (*n* = 78 female, *M_age_* = 41.2 years, *SD_age_* = 13.2 years) matched by age, sex, and site were drawn from the baseline assessment of the Marburg-Münster Affective Disorders Cohort Study (MACS) [53] (Table 1). Participants aged 18–65 were recruited in Münster and Marburg through local psychiatric hospitals and newspaper advertisements. The study was approved by the ethics committees of University of Marburg (AZ: 07/14) and Münster (2014-422-b-S), in accordance with the Declaration of Helsinki. All participants gave written informed consent and received financial compensation. Exclusion criteria included usual magnetic resonance imaging (MRI) contraindications, head trauma, and past and current neurological, cardiovascular, or other serious illnesses, and current substance dependence. HC had no lifetime mental disorder and no current intake of psychotropic medication. Diagnoses or lack thereof were assessed using the Structured Clinical Interview for DSM-IV-TR for Axis I disorder (SCID-I) [54], conducted by trained personnel.

**Table 1.** Demographic and clinical characteristics of BD patients and HC

	BD (*n* = 153)	HC (*n* = 153)	Group comparison	
			Test-statistic	*p*-value
Demographics				
Age	41.0 ± 12.0	41.2 ± 13.2	0.163	.871^+^
Biological sex (f/m)	79/74	78/75	0	1^$^
Site (Marburg/Münster)	58/95	61/92	0.055	.815
Questionnaires				
HDRS scores	7.84 ± 7.02	1.23 ± 1.61	−11.35	<.001^+^
YMRS scores	3.75 ± 5.51	0.78 ± 1.66	−6.36	<.001^+^
GAF scores	61.9 ± 12.6 (*n* = 151)	90.4 ± 7.9 (*n* = 151)	23.56	<.001^+^
Psychiatric medication				
Medication load index	2.72 ± 2.06	*n*/a	*n*/a	*n*/a
None (yes/no)	23/130	*n*/a	*n*/a	*n*/a
Antidepressants (yes/no)	63/90	*n*/a	*n*/a	*n*/a
Antipsychotics (yes/no)	82/71	*n*/a	*n*/a	*n*/a
Anticonvulsives (yes/no)	44/109	*n*/a	*n*/a	*n*/a
Lithium (yes/no)	46/107	*n*/a	*n*/a	*n*/a
Clinical characteristics				
Bipolar subtype (BD1/BD2)	89/64	*n*/a	*n*/a	*n*/a
Course of the disease (single episode with good remission/good remission between episodes/partial remission between episodes/persistent chronic illness)*	2/83/56/12	*n*/a	*n*/a	*n*/a
Remission status (acute/partly−/fully remitted)	69/37/39 (*n* = 145)	*n*/a	*n*/a	*n*/a
Age of onset	23.9 ± 11.1	*n*/a	*n*/a	*n*/a
Lifetime psychiatric comorbidity (yes/no)	63/90	*n*/a	*n*/a	*n*/a
Number of depressive episodes	7.34 ± 7.43 (*n* = 149)	*n*/a	*n*/a	*n*/a
Duration of depressive episodes (months)	46.7 ± 67.4 (*n* = 132)	*n*/a	*n*/a	*n*/a
Number of (hypo-)manic episodes	5.49 ± 7.52	*n*/a	*n*/a	*n*/a
First manic episode/multiple manic episodes	40/113	*n*/a	*n*/a	*n*/a
Duration of (hypo-)manic episodes (months)	16.5 ± 31.6 (*n* = 133)	*n*/a	*n*/a	*n*/a
Number of inpatient treatments	3.69 ± 3.12 (*n* = 150)	*n*/a	*n*/a	*n*/a
Duration of inpatient treatments (weeks)	32.1 ± 32.0 (*n* = 148)	*n*/a	*n*/a	*n*/a
Duration of sick leave (months)	20.1 ± 48.7 (*n* = 138)	*n*/a	*n*/a	*n*/a
Duration of retirement (months)	24.4 ± 55.3 (*n* = 107)	*n*/a	*n*/a	*n*/a

*Note*: Data are mean ± SD or frequencies. BD, bipolar disorder; HC, healthy controls; HDRS, 21-item Hamilton Depression Rating Scale; GAF, General Assessment of Functioning Scale; n/a, not applicable; YMRS, Young Mania Rating Scale. +Calculated using the paired two-tailed Student’s *t* test. $Calculated using the *χ*^2^ test. *Derived from Item 90 of the Operational Criteria (OPCRIT) Checklist for Affective and Psychotic Illness.

### Clinical characteristics

During the interview, patients provided retrospective self-reports on their previous course of illness, including the number of manic episodes and quality of remission between previous episodes, assessed with the Operational Criteria (OPCRIT) Checklist for Affective and Psychotic Illness [55]. Patients were categorized as “single episode with good remission,” “multiple episodes with good remission between episodes,” “multiple episodes with partial remission between episodes” or “persistent chronic illness.” For analysis, the first two categories were combined into “good remission” (BD_rem_), while the latter two were grouped under “chronic course” (BD_chron_) to ensure balanced group sizes and simplify interpretability by using conceptually meaningful groups. The presence and severity of acute depressive and manic symptoms were assessed using the 21-item Hamilton Depression Rating Scale (HDRS) [56] and the Young Mania Rating Scale (YMRS) [57], respectively. The level of functioning was assessed via the Global Assessment of Functioning (GAF) [58]. The type and amount of current medication were assessed and summarized in one medication load index, as described earlier [59] (Supplement 1).

### DTI data acquisition and pre-processing

DTI data were acquired with two 3 T whole body MR scanners (Marburg: Tim Trio, Siemens, Erlangen, Germany; Münster: Prisma fit, Siemens, Erlangen, Germany). All images were thoroughly quality controlled according to the published protocol of the MACS study [60]. Due to changes of the body coil (BC) and gradient coil (GC) in the MRI scanner in Marburg, we controlled for four different scanner settings, including three dummy-coded variables (BC and GC pre change, BC post and GC pre change, BC and GC post change) with Münster as the reference category as covariates in all analyses, as previously recommended [60].

Preprocessing, quality assurance, and analyses were performed in FSL6.0.1 (http://fsl.fmrib.ox.ac.uk/fsl/fslwiki/) [61–63] and followed published protocols described elsewhere [38, 60]. For details on DTI acquisition, quality assurance, preprocessing, and analysis, see Supplement 2. DTI metrics (fractional anisotropy (FA), mean diffusivity (MD), radial diffusivity (RD), and axial diffusivity (AD)) were calculated based on the computed diffusion tensor. MD, RD, and AD were analyzed in the same way as FA (Supplement 3), but we focus on FA as the most widely employed DTI measure. It captures water diffusion directionality on a scale from 0 (isotropic diffusion) to 1 (completely anisotropic diffusion) and is hypothesized to reflect fiber density and degree of myelination [64, 65].

### Statistical analyses

Demographic, clinical, and cognitive data were analyzed in R Studio (version 4.2.2; R Core Team, 2022).

#### Analyses of DTI data

The DTI data were analyzed using the tract-based spatial statistics (TBSS) technique implemented in FSL, which reduces partial volume effects and misalignment during registration [66]. The analyses were adjusted for alpha inflation using the non-parametric permutation tests implemented in FSL *randomize* [67]. Threshold-Free Cluster Enhancement (TFCE) was applied with 5000 permutations per test [68] and corrected for familywise error (FWE; *p* < 0.05). Additionally, FDR correction [69] was applied to all post-hoc tests. In all analyses, age, sex, total intracranial volume (TIV), and the scanner variables were included as covariates. All analyses that yielded significant results in the BD patient groups were further checked for robustness by including additional covariates (current symptom severity, age of onset, lifetime comorbidity, BD subtype, and medication; Supplement 5). To investigate WM integrity in association with disease progression of BD, we used three different approaches:

**Analysis 1: Number of manic episodes.** First, we categorized patients based on the lifetime number of manic episodes: *N* = 40 patients had experienced only one (hypo-)manic episode in their lives (BD_first_), *n* = 113 patients had experienced two or more manic episodes (BD_multiple_) (Table 2). A one-factorial analysis of covariance (ANCOVA) was performed with group as the independent variable (HC vs. BD_first_ vs. BD_multiple_) and FA as dependent variable (*F*-test), followed by post-hoc paired *t*-tests.

**Table 2.** Demographic and clinical characteristics of BD patients depending on the number of manic episodes or the quality of remission between previous episodes

	BD_first_ (*n* = 40)	BD_multiple_ (*n* = 113)	Group comparison		BD_rem_ (*n* = 85)	BD_chron_ (*n* = 68)	Group comparison	
			Test-statistic	*p*-value			Test-statistic	*p*-value
Demographics								
Age	38.38 ± 12.64	41.93 ± 11.73	−1.557	.124^+^	41.4 ± 12.1	40.5 ± 12.0	0.472^+^	.637^+^
Biological sex (f/m)	20/20	59/54	0.003	.955^$^	47/38	32/36	0.72267^$^	.395^$^
Site (Marburg/Münster)	13/27	45/68	0.398	.528^$^	34/51	24/44	0.184	.668^$^
Questionnaires								
HDRS scores	7.93 ± 6.31	7.81 ± 7.29	0.099	.922^+^	5.92 ± 5.93	10.2 ± 7.56	−3.85^+^	<.001^+^
YMRS scores	3.18 ± 3.84	3.95 ± 6.0	−0.932	.354^+^	2.85 ± 4.65	4.87 ± 6.29	−2.21^+^	.029^+^
GAF scores	66.4 ± 11.4	60.2 ± 12.6 (*n* = 111)	2.855	.006^+^	66.2 ± 12.5 (*n* = 84)	56.4 ± 10.5 (*n* = 67)	5.22^+^	<.001^+^
Psychiatric medication								
Medication load index	2.08 ± 1.56	2.95 ± 2.17	1717	.022^~^	2.38 ± 1.94	3.15 ± 2.13	2286.5	.025^~^
None (yes/no)	6/34	17/96	0	1^$^	17/68	6/62	2.87^$^	.090^$^
Antidepressants (yes/no)	19/21	44/69	0.576	.448^$^	35/50	28/40	0^$^	1^$^
Antipsychotics (yes/no)	17/23	65/48	2.11	.146^$^	41/44	41/27	1.75^$^	.186^$^
Anticonvulsives (yes/no)	6/34	38/75	4.136	.042^$^	17/68	27/41	6.23^$^	.013^$^
Lithium (yes/no)	11/29	35/78	0.045	.833^$^	28/57	18/50	0.476^$^	.490^$^
Clinical characteristics lifetime								
Quality of remission (BD_rem_/BD_chron_)	26/14	59/54	1.473	.225^$^	-	-	-	-
Number of manic episodes (BD_first_/BD_multiple_)	-	-	-	-	26/59	14/54	1.473	.225^$^
Bipolar subtype (BD1/BD2)	18/22	71/42	3.163	.075^$^	52/33	37/31	0.46^$^	.498^$^
Remission status (acute/partly−/fully remitted)	18/12/9 (*n* = 39)	51/25/30 (*n* = 106)	0.889	.641^$^	29/18/32 (*n* = 79)	40/19/7 (*n* = 66)	16.78^$^	<.001^$^
Age of Onset	25.4 ± 10.8	23.4 ± 11.2	1.028	.307^+^	23.2 ± 11.0	24.6 ± 11.3	−0.729^+^	.467^+^
Lifetime psychiatric comorbidity (yes/no)	21/19	42/71	2.269	.132^$^	33/52	30/38	0.246^+^	.62^$^
Number of depressive episodes	4.11 ± 3.24 (*n* = 38)	8.44 ± 8.11 (*n* = 111)	−4.648	<.001^+^	6.24 ± 5.56 (*n* = 83)	8.71 ± 9.12 (*n* = 66)	−1.93^+^	.056^+^
Duration of depressive episodes (months)	19.8 ± 18.7 (*n* = 37)	57.1 ± 76.2 (*n* = 95)	−4.446	<.001^+^	30.7 ± 39.0(*n* = 74)	67.0 ± 88.0 (*n* = 58)	−2.92^+^	.005^+^
Number of (hypo-)manic episodes	1.0 ± 0.0	7.08 ± 8.19	−7.89	<.001^+^	5.19 ± 8.45 (*n* = 85)	5.87 ± 6.22 (*n* = 68)	−0.573^+^	.568^+^
Duration of (hypo-)manic episodes (months)	4.64 ± 8.3 (*n* = 37)	21.0 ± 35.8 (*n* = 96)	−4.202	<.001^+^	10.2 ± 16.8 (*n* = 76)	24.8 ± 43.0 (*n* = 57)	−2.435^+^	.017^+^
Number of inpatient treatments	2.85 ± 2.13	3.99 ± 3.36	−2.449	.016^+^	3.27 ± 2.92 (*n* = 82)	4.21 ± 3.3 (*n* = 68)	−1.824^+^	.070^+^
Duration of inpatient treatments (weeks)	22.5 ± 24.5 (*n* = 39)	35.6 ± 33.74 (*n* = 109)	−2.585	.011^+^	27.8 ± 26.1 (*n* = 82)	37.5 ± 37.7 (*n* = 66)	−1.779^+^	.078^+^
Duration of sick leave (months)	10.6 ± 10.2 (*n* = 36)	23.4 ± 56.0 (*n* = 102)	−2.207	.029^+^	20.1 ± 59.4 (*n* = 77)	20.0 ± 30.7 (*n* = 61)	0.017^+^	.987^+^
Duration of retirement (months)	13.5 ± 40.8 (*n* = 26)	27.9 ± 58.1(*n* = 81)	−1.398	.167^+^	4.60 ± 20.0 (*n* = 54)	44.6 ± 70.8 (*n* = 53)	−3.96^+^	<.001^+^

*Note*: Data are mean ± SD or frequencies. BD = bipolar disorder, BD_first_ = BD patients who experienced only one hypomanic or manic episode in their lives, BD_multiple_ = BD patients who already experienced two or more manic episodes, BD_rem_ = BD patients who experienced a good remission between previous episodes in their lives, BD_chron_ = BD patients who achieved only partial remission between episodes or had already developed a chronic course (derived from Item 90 of the Operational Criteria (OPCRIT) Checklist for Affective and Psychotic Illness, which assesses the previous course of the illness), HDRS = 21-item Hamilton Depression Rating Scale, GAF = General Assessment of Functioning Scale, YMRS=Young Mania Rating Scale. +Calculated using the paired two-tailed Student’s t test. $Calculated using the χ^2^ test. ~Calculated using the Mann–Whitney-U-Test.

**Analysis 2: Quality of remission.** Second, the patients were categorized into two groups based on their previous quality of remission as assessed using the OPCRIT checklist for affective and psychotic disorders: *N* = 85 patients (BD_rem_) experienced a good remission between previous episodes in their lives. In contrast, *n* = 68 patients achieved only partial remission between episodes or had already developed a chronic course (BD_chron_) (Table 2). Again, a one-factorial ANCOVA was performed with group as the independent variable (HC vs. BD_rem_ vs. BD_chron_) and FA as the dependent variable (*F*-test), followed by post-hoc paired *t*-tests.

**Analysis 3a: Level of functioning.** Finally, to investigate whether the level of functioning was related to WM microstructure, a linear regression model was used to calculate an association between the GAF score and FA. As outlined, we focus on interepisodic functional levels. Therefore, only BD patients who were (partially) remitted at the time of measurement were included in this analysis (*n* = 75, Supplementary Table S1). For completeness, the analysis was also conducted on the full sample (Supplement 4).

## Results

### Analysis 1. WM microstructural differences among HC, BD_first_, and BD_multiple_

**Figure 1.**
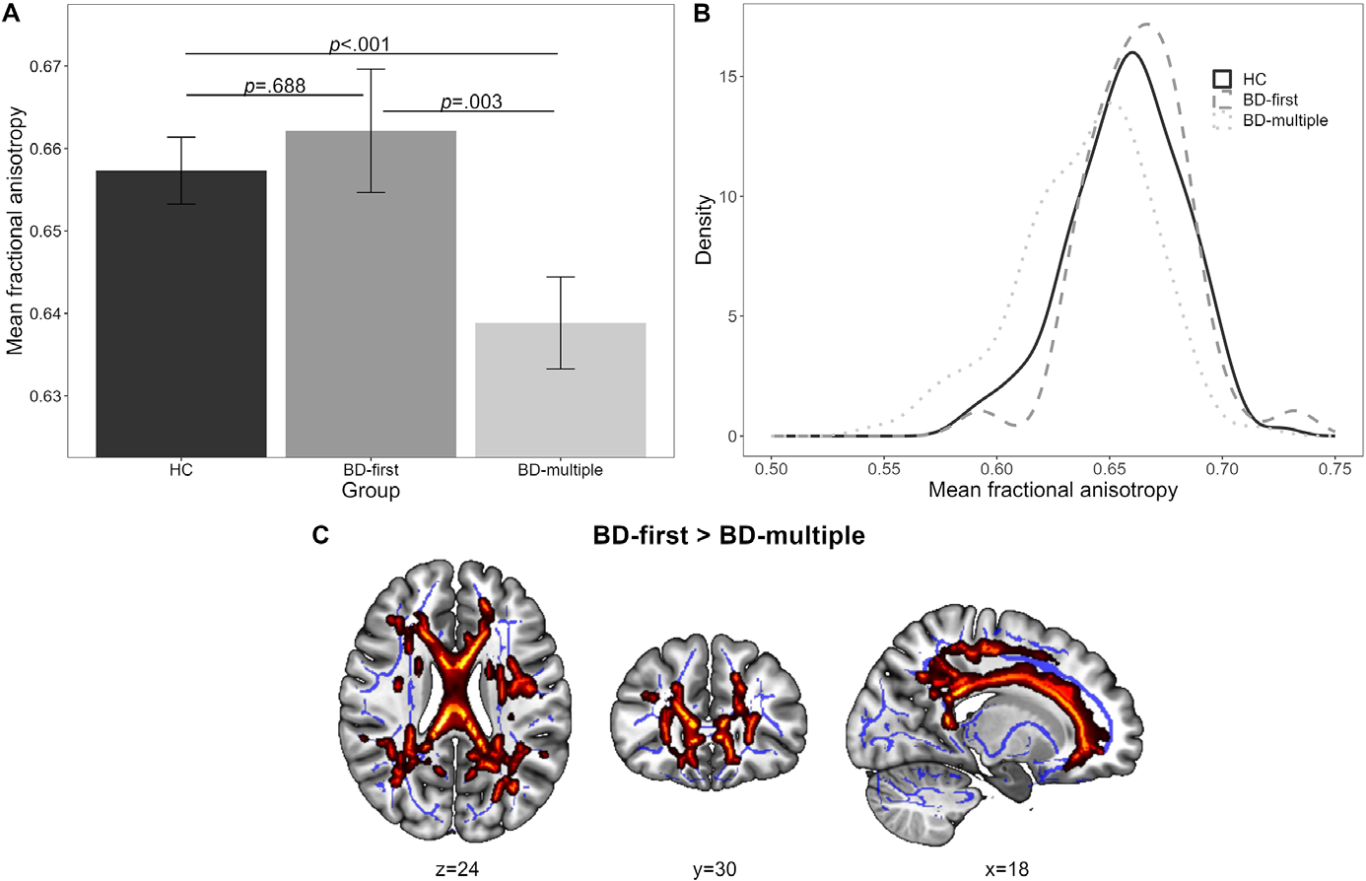
Differences in FA between HC and BD categorized into stages based on the number of manic episodes. Note. (A) Mean fractional anisotropy (FA) across healthy controls (HC), patients with bipolar disorder (BD) who have only experienced a first manic episode (BD-first), and patients with BD who have already experienced multiple manic episodes (BD-multiple). The mean FA value was obtained from FA values of all the voxels that showed a significant main effect of diagnosis (*ptfce-FWE* < 0.05). Error bars represent 95% confidence intervals. *p-values* were obtained from pairwise post hoc *t*-contrasts. (B) Density estimation plots of FA values for the three groups: HC, BD-first, and BD-multiple. (C) Higher FA in BD-first compared with BD-multiple. Statistically significant clusters from the post-hoc *t*-contrast are displayed on the MNI152 template using MRIcroGL (version 1.2). Highlighted areas represent voxels (using FSL’s ‘fill’ command for better visualization), where significant differences between groups (*p*_tfce-FWE_ < 0.05) were detected. MNI = Montreal Neurological Institute.

There was a significant main effect of group in FA (*F*-contrast: *p*_tfce-FWE_ = 0.001, total *k* = 6843 voxels in seven clusters, Figure 1A and B, Supplementary Table S2), which was further examined with pairwise comparisons: These revealed significantly lower FA values in BD_multiple_ compared with HC in one large bilateral cluster (*d* = 0.21, *p*_tfce-FWE_ < 0.001, *k* = 45,480 voxels) as well as compared with BD_first_ (*d* = 0.30, *p*_tfce-FWE_ = 0.003, *k* = 23,478 voxels in seven clusters, Figure 1C), with both differences remaining significant after FDR correction (*p* = 0.003 and *p* = 0.005). In contrast, BD_first_ patients did not show significantly different FA values compared with HC (*p*_tfce-FWE_ = 0.688). Both effects were mainly localized in the corpus callousum, the corona radiata, and the superior longitudinal fasciculus (Supplementary Table S3). The difference between the two BD groups remained significant even when adjusting for additional clinical characteristics (Supplementary Table S2). There also emerged a significant main effect of group for RD and MD, reflected by significantly higher scores for BD_multiple_ compared with HC and BD_first_. No effects were found for AD (Supplement 3).

### Analysis 2. WM microstructural differences among HC, BD_rem,_ and BD_chron_

**Figure 2.**
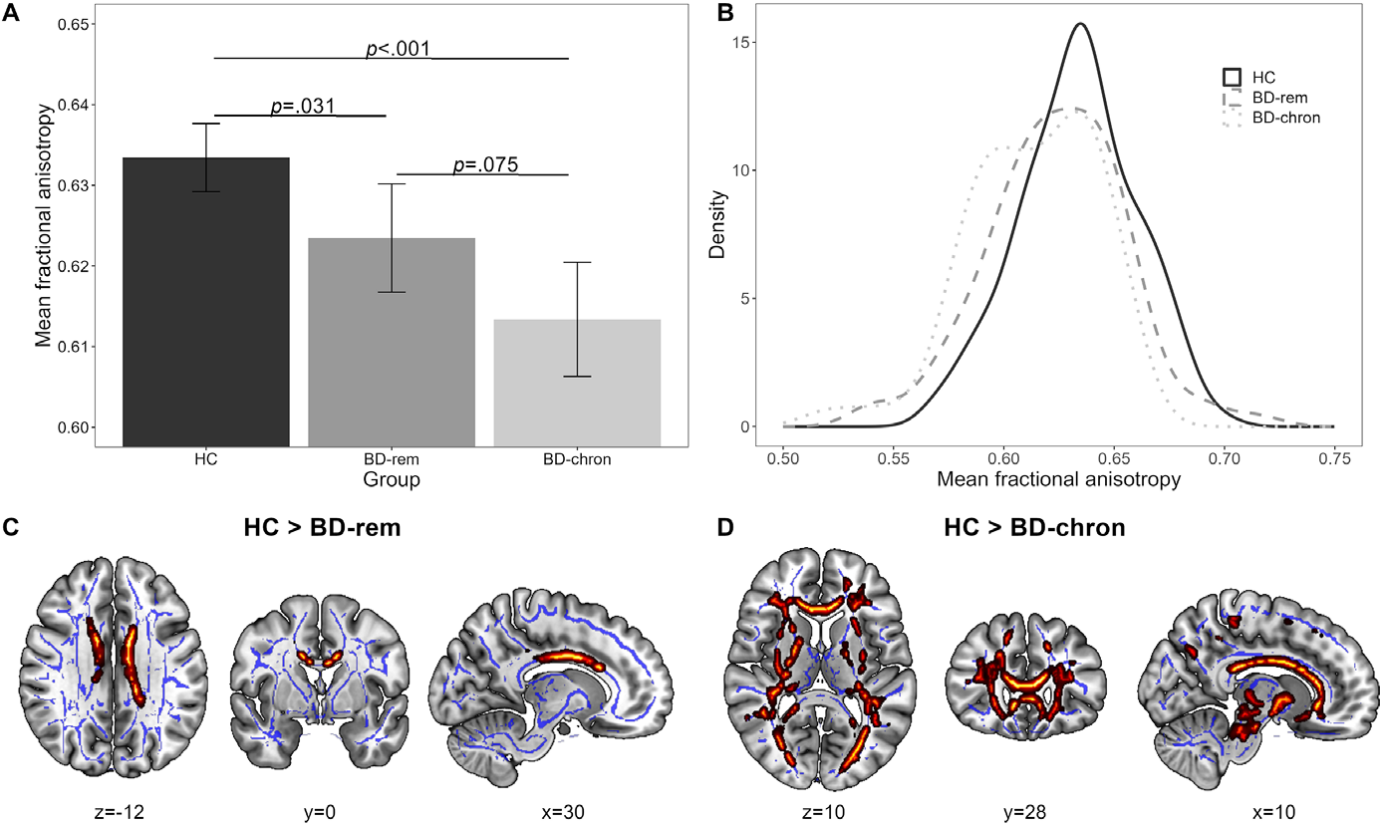
Differences in FA between HC and BD categorized into stages based on the quality of remission between episodes*. Note*: (A) Mean fractional anisotropy (FA) across healthy controls (HC), patients with bipolar disorder (BD) achieving stable remission between episodes (BD-rem), and patients with BD achieving partial or no remission between episodes (BD-chron). The mean FA value was obtained from FA values of all the voxels that showed a significant main effect of diagnosis (*ptfce-FWE* < 0.05). Error bars represent 95% confidence intervals. *p-values* were obtained from pairwise post hoc *t*-contrasts. (B) Density estimation plots of FA values for the three groups HC, BD-rem, and BD-chron. (C-D) Higher FA in HC compared with BD-rem (c) or BD-chron (d). Statistically significant clusters from the post-hoc *t*-contrasts are displayed on the MNI152 template using MRIcroGL (version 1.2). Highlighted areas represent voxels (using FSL’s “fill” command for better visualization), where significant differences between groups (*p*_tfce-FWE_ < 0.05) were detected. MNI, Montreal Neurological Institute.

When categorizing BD patients based on their previous remission quality, the *F*-contrast again revealed a significant main effect of group in FA (*p*_tfce-FWE_ = 0.005, total *k* = 1764 voxels in five clusters, Figure 2A and B, Supplementary Table S2). Pairwise post-hoc *t*-contrasts revealed significantly higher FA values for HC compared with BD_chron_ (*d* = 0.26, *p*_tfce-FWE_ < 0.001, total k = 39,297 voxels in one cluster, Figure 2D)_,_ as well as BD_rem_, albeit less pronounced (*d* = 0.33, *p*_tfce-FWE_ = 0.031, total k = 1426 voxels in four clusters, Figure 2C). Both effects remained significant after FDR correction (*p* = 0.003 and *p* = 0.047) and were mainly localized in the corpus callousum and the corona radiata, whereas the comparison between HC and BD_chron_ also included the internal and external capsule, posterior thalamic radiation and superior longitudinal fasciculus (Supplementary Table S3). BD_chron_ showed lower FA values compared to BD_rem_, although this difference did not reach statistical significance (*p*_tfce-FWE_ = 0.075). There also emerged a significant main effect of group for RD, reflected by significantly higher scores for BD_chron_ compared with HC and BD_rem_. No effects were found for MD and AD (Supplement 3).

### Analysis 3. Association between GAF scores and WM microstructure in remitted BD patients

**Figure 3.**
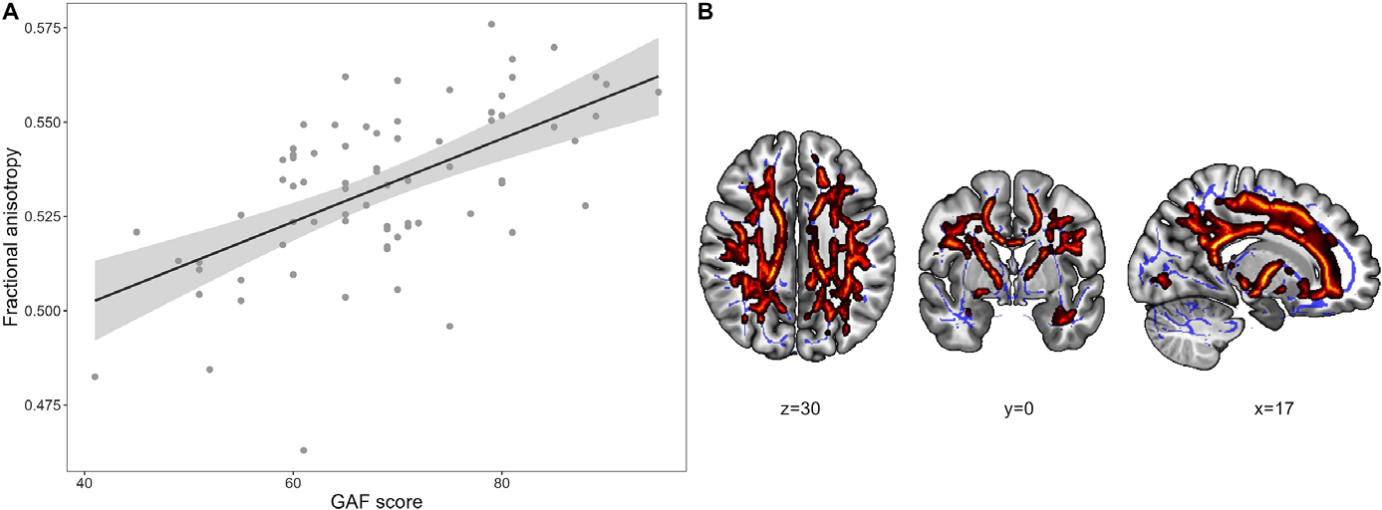
Positive association between GAF scores and FA in euthymic BD patients*. Note*: (A) Scatterplot depicting the cross-sectional association between GAF scores and fractional anisotropy (FA) in euthymic patients with bipolar disorder (BD). Each datapoint represents one participant. Lines and shaded areas indicate the mean association between FA and GAF scores as well as the confidence intervals. The FA value was obtained from the FA values of all the voxels that showed a significant positive association (*ptfce-FWE* < 0.05). (B) Statistically significant clusters from the positive association effect are displayed on the MNI152 template using MRIcroGL (version 1.2). Highlighted areas represent voxels (using FSL’s “fill” command for better visualization), where a significant association between variables (*p*_tfce-FWE_ < 0.05) was detected. MNI = Montreal Neurological Institute.

As explained, we focus on interepisodic functional level, reporting results from euthymic subjects. The linear regression analysis investigating an association between the GAF score and FA in euthymic BD patients yielded a significant positive association (*p_tfce-FWE_* < 0.001, one cluster with *k* = 43,114 voxels, Figure 3), which remained significant when additionally controlling for clinical characteristics (Supplementary Table S2). A negative association was found for MD and RD, while no effect emerged for AD (Supplement 3). The effects were mainly localized in the corpus callosum, corona radiata, and superior longitudinal fasciculus (Supplementary Table S3).

## Discussion

This study was the first to comprehensively investigate whether disease progression in BD is reflected in WM integrity, using three approaches derived from staging models. Overall, our results support our hypothesis that early and late stages of BD differ in WM microstructure, as we found higher WM integrity in patients with fewer manic episodes and in those with higher levels of functioning outside of acute episodes. Although no clear group differences emerged when remission between episodes was used as a progression criterion, these results provide initial evidence that WM alterations may relate to illness progression.

Our finding of lower WM integrity in patients with a first manic episode compared to patients with multiple manic episodes aligns with other studies [43, 70], which reported localized effects in the corpus callosum. Although this fiber tract was also found to be centrally affected in all our analyses, we observed more global and widespread WM microstructure impairments throughout the brain, involving various projection, association, and commissural pathways. As both the significance of this widespread effect and the specific role of the corpus callosum have been addressed in our previous research on BD [38, 40], they will not be further discussed here. Although our remission-based approach showed no significant differences between early and late stages in direct comparisons, patients with partial or no remission showed widespread WM alterations compared to HC, whereas patients with stable remission differed from HC only in small local clusters. This locality versus globality also indicates greater WM impairments in later stages. The positive correlation between WM microstructure and global functioning, as measured by the GAF score, in remitted BD patients highlights the clinical significance of these WM impairments. Although we are the first to investigate this specific relationship, our finding aligns with studies linking GAF and WM volume [71, 72]. Cognitive impairments or residual depressive symptoms – key predictors of euthymic functional impairment [73–78] – may mediate this correlation. Cognitive deficits in particular have been associated with WM microstructural impairment in BD patients [79]. Overall, our findings on functioning should be interpreted with caution, as interepisodic functioning served more as an indirect measure of disease progression. Furthermore, impairments in this domain can occur independently of the disease – even in HC – and we lack data on the patients‘premorbid functioning.

Regarding the differences between our two categorical approaches, it can first be assumed that the episode-based categorization rather measures the effects of repeated acute stress, whereas the second approach is more likely to capture the effects of chronic stress, caused by incomplete remission with persistent residual symptoms, which seems to be less clearly associated with WM alterations. Moreover, the first approach differentiates more in earlier stages, while the second approach focuses more on later stages. Patients were more likely to be classified in the latter group in the first approach than in the second, which is also reflected in the differences in the respective group sizes. The fact that significant differences were found within BD patients using the first approach, but not the second, may lend support to the hypothesis discussed in the literature that WM abnormalities tend to emerge earlier in the course of the disease, while later stages no longer lead to significant changes [80–83].

All our analyses underwent comprehensive robustness checks, accounting for clinical features previously shown to influence WM integrity, including pharmacological treatment [39, 84], BD subtype [40], or age of onset [39, 85]. We therefore conclude that the alterations identified are independently associated with the variables used to assess disease progression. However, since this is a cross-sectional study, our findings can be interpreted in two ways: either as an indication of neuroprogression, that is, cumulative changes due to repeated experiences of episodes or symptoms [e.g. 43, 47, 70], or as trait-like characteristics of clinical subtypes that already differ a priori in the studied characteristics (i.e. patients with pronounced WM alterations are also more likely to reach later stages) [2, 32]. Both interpretations are supported by the broader research on cognitive deficits in BD and seem plausible, especially given the lack of longitudinal DTI studies. Importantly, the interpretation of clinical subtypes does not rule out the presence of neuroprogression in certain subgroups, but rather reflects the heterogeneity of BD [16]. This heterogeneity results not only from different forms of progression but also from factors such as BD subtypes, predominant polarity, age of onset, response to treatment, psychotic features, suicide attempts, or rapid cycling [16, 86, 87]. Neurobiological differences between such subtypes support this approach of clinical subtypes [86–91], such as our prior finding of more severe WM impairments in BD subtype I versus II [40]. Moreover, our categorization by manic episodes did not consider the possible contribution of depressive episodes, which raises the factor of predominant manic polarity as an explanation for the observed differences as well as the discrepancies between approaches. This is illustrated by the fact that some patients with only one manic episode were classified as BD_chron_ (Table 1), likely due to a disease course dominated by depressive episodes. There are some studies suggesting that a predominant manic polarity may be associated with more severe progressive impairment compared to a predominant depressive polarity [92, 93], possibly due to the frequent occurrence of psychotic symptoms during mania [94] and the use of certain medications such as antipsychotics or anticonvulsants [39, 85, 95].

The findings of this study should be interpreted with certain limitations in mind. One crucial limitation of our study is its cross-sectional design, which does not allow causal inferences. The question of neuroprogression is inherently a longitudinal one, which cannot be fully answered by cross-sectional studies. Future longitudinal studies in BD are therefore urgently needed. In addition, one limitation lies in the use of simplified staging approaches. Instead of employing a detailed staging model, we relied on broad dichotomous classifications, which miss subtle differences in disease progression as each category encompasses a wide range of clinical severity. While this approach aligns with the broader recommendations of the ISBD [50], a more nuanced method combining multiple variables would have been more sound. In the future, detailed clinically validated staging models may provide better insight into the associated neurobiological changes [5]. Furthermore, even though we made several robustness checks, not all possible influences on the results could be excluded, as our sample was very heterogeneous. For example, previous disease history, such as depressive episodes prior to diagnosis, specific comorbidities, or previous psychopharmacological treatments, may have undetected influences. Finally, the use of more advanced tractography techniques could provide a more detailed and hypothesis-driven investigation across specific WM pathways, thus usefully complementing the whole-brain voxel-wise analysis that we performed via TBSS [96].

In conclusion, our study provides important insights into the relationship between WM microstructure and the clinical course of BD. Patients in advanced stages showed lower WM integrity compared to HC and, partially, to patients in earlier stages, and lower WM integrity was associated with poorer functioning. Due to the cross-sectional design, the results leave open whether they are truly indicative of a progressive course. Nevertheless, our findings highlight the clinical relevance of WM alterations. They not only advance our understanding of the biological mechanisms underlying disease progression but may also inform future clinical practice. Incorporating patterns of WM alterations associated with early versus late stages into clinical assessments could enable more accurate evaluation of disease progression, earlier identification of patients at high risk for rapid progression and functional impairment, and support the implementation of personalized, stage-specific treatment strategies. Although further research is needed before these findings can be directly applied in clinical practice, integrating WM alterations into refined and validated staging models could increase their diagnostic and prognostic utility while keeping in mind the complexity and heterogeneity of BD.

## Supporting information

10.1192/j.eurpsy.2025.10105.sm001 Thiel et al. supplementary material Thiel et al. supplementary material

## Data Availability

The data that support the findings of this study are available from the corresponding author, upon reasonable

## Supplementary material

The supplementary material for this article can be found at http://doi.org/10.1192/j.eurpsy.2025.10105.

## References

[1] Grewal S, McKinlay S, Kapczinski F, Pfaffenseller B, Wollenhaupt-Aguiar B. Biomarkers of neuroprogression and late staging in bipolar disorder: A systematic review. Aust N Z J Psychiatry [Internet]. 2023;57(3):328–43. 10.1177/00048674221091731. 35403455 PMC9950598

[2] Passos IC, Mwangi B, Vieta E, Berk M, Kapczinski F. Areas of controversy in neuroprogression in bipolar disorder. Acta Psychiatr Scand [Internet]. 2016;134(2):91–103. [Accessed 19 Dec 2023]. Available from: https://onlinelibrary.wiley.com/doi/abs/10.1111/acps.12581. 27097559 10.1111/acps.12581

[3] Berk M, Conus P, Lucas N, Hallam K, Malhi GS, Dodd S, et al. Setting the stage: from prodrome to treatment resistance in bipolar disorder. Bipolar Disorders [Internet]. 2007;9(7):671–8. [Accessed 3 Nov 2023]. Available from: https://onlinelibrary.wiley.com/doi/abs/10.1111/j.1399-5618.2007.00484.x. 17988356 10.1111/j.1399-5618.2007.00484.x

[4] Kapczinski F, Dias VV, Kauer-Sant’Anna M, Frey BN, Grassi-Oliveira R, Colom F, et al. Clinical implications of a staging model for bipolar disorders. Expert Rev Neurother. 2009;9(7):957–66. 19589046 10.1586/ern.09.31

[5] Berk M, Post R, Ratheesh A, Gliddon E, Singh A, Vieta E, et al. Staging in bipolar disorder: From theoretical framework to clinical utility. World Psychiatry. 2017; 16(3):236–44. 28941093 10.1002/wps.20441 PMC5608827

[6] Cosci F, Fava GA. Staging of mental disorders: Systematic review. Psychother Psychosom. 2013;82(1):20–34. 23147126 10.1159/000342243

[7] Berk M. Neuroprogression: Pathways to progressive brain changes in bipolar disorder. Int J Neuropsychopharmacol. 2009;12(4): 441–5. 18922203 10.1017/S1461145708009498

[8] van der Markt A, Klumpers UMH, Dols A, Draisma S, Boks MP, van Bergen A, et al. Exploring the clinical utility of two staging models for bipolar disorder. Bipolar Disorders [Internet]. 2020 ;22(1):38–45. [Accessed 3 Nov 2023]. Available from: https://onlinelibrary.wiley.com/doi/abs/10.1111/bdi.12825. 31449716 10.1111/bdi.12825 PMC7065163

[9] van der Markt A, Klumpers UM, Draisma S, Dols A, Nolen WA, Post RM, et al. Testing a clinical staging model for bipolar disorder using longitudinal life chart data. Bipolar Disorders [Internet]. 2019;21(3):228–34. [Accessed 1 Oct 2024]. Available from: https://onlinelibrary.wiley.com/doi/abs/10.1111/bdi.12727. 30447123 10.1111/bdi.12727 PMC6590317

[10] Rosa AR, Magalhães PVS, Czepielewski L, Sulzbach MV, Goi PD, Vieta E, et al. Clinical staging in bipolar disorder: focus on cognition and functioning. J Clin Psychiatry [Internet]. 2014;75(5):e450–6. [Accessed 2 Nov 2023]. Available from: https://www.psychiatrist.com/jcp/clinical-staging-bipolar-disorder-focus-cognition. 24922497 10.4088/JCP.13m08625

[11] Macellaro M, Girone N, Cremaschi L, Bosi M, Cesana BM, Ambrogi F, et al. Staging models applied in a sample of patients with bipolar disorder: results from a retrospective cohort study. J Affect Disord [Internet]. 2023;323:452–60. [Accessed 8 Oct 2024]. Available from: https://www.sciencedirect.com/science/article/pii/S0165032722013416. 36455717 10.1016/j.jad.2022.11.081

[12] Tatay-Manteiga A, Correa-Ghisays P, Cauli O, Kapczinski FP, Tabarés-Seisdedos R, Balanzá-Martínez V. Staging, neurocognition and social functioning in bipolar disorder. Front Psychiatr [Internet]. 2018;9:709. [Accessed 2 Nov 2023]. Available from: https://www.frontiersin.org/articles/10.3389/fpsyt.2018.00709. 10.3389/fpsyt.2018.00709 PMC6305735 30618879

[13] Cremaschi L, Macellaro M, Girone N, Bosi M, Cesana BM, Ambrogi F, et al. The progression trajectory of bipolar disorder: results from the application of a staging model over a ten-year observation. J Affect Disord. 2024;362:186–93. 38944295 10.1016/j.jad.2024.06.094

[14] Lee Y, Lee D, Jung H, Cho Y, Baek JH, Hong KS. Heterogeneous early illness courses of Korean patients with bipolar disorders: Replication of the staging model. BMC Psychiatry [Internet]. 2022;22:684. [Accessed 18 Dec 2023]. Available from: https://bmcpsychiatry.biomedcentral.com/articles/10.1186/s12888-022-04318-y. 36333702 10.1186/s12888-022-04318-y PMC9636704

[15] Magalhães PV, Dodd S, Nierenberg AA, Berk M. Cumulative morbidity and prognostic staging of illness in the systematic treatment enhancement program for bipolar disorder (STEP-BD). Aust N Z J Psychiatry*N*. 2012;46(11):1058–67. 10.1177/0004867412460593 23015748

[16] Alda M, Kapczinski F. Staging model raises fundamental questions about the nature of bipolar disorder. J Psychiatry Neurosci [Internet]. 2016;41(5):291–3. [Accessed 19 Dec 2023]. Available from: https://www.ncbi.nlm.nih.gov/pmc/articles/PMC5008917/. 27575857 10.1503/jpn.160151 PMC5008917

[17] Malhi GS, Rosenberg DR, Gershon S. Staging a protest! Bipolar Disorders [Internet]. 2014;16(7):776–9. [Accessed 10 Oct 2024]. Available from: https://onlinelibrary.wiley.com/doi/abs/10.1111/bdi.12254. 25195710 10.1111/bdi.12254

[18] Strejilevich SA, Samamé C, Martino DJ. The trajectory of neuropsychological dysfunctions in bipolar disorders: a critical examination of a hypothesis. J Affect Disord 2015;175:396–402. 25678172 10.1016/j.jad.2015.01.018

[19] Martino DJ, Igoa A, Marengo E, Scápola M, Strejilevich SA. Longitudinal relationship between clinical course and neurocognitive impairments in bipolar disorder. J Affect Disord. 2018;225:250–5. 28841488 10.1016/j.jad.2017.08.011

[20] Flaaten CB, Melle I, Bjella T, Engen MJ, Åsbø G, Wold KF, et al. Long-term course of cognitive functioning in bipolar disorder: A ten-year follow-up study. Bipolar Disorders [Internet]. 2024;26(2):136–47. [Accessed 10 Oct 2024]. Available from: https://onlinelibrary.wiley.com/doi/abs/10.1111/bdi.13364. 37356974 10.1111/bdi.13364

[21] Santos JL, Aparicio A, Bagney A, Sánchez-Morla EM, Rodríguez-Jiménez R, Mateo J, et al. A five-year follow-up study of neurocognitive functioning in bipolar disorder. Bipolar Disord [Internet]. 2014;16(7):722–31. [Accessed 21 Nov 2024]. Available from: https://onlinelibrary.wiley.com/doi/abs/10.1111/bdi.12215. 24909395 10.1111/bdi.12215

[22] Mora E, Portella MJ, Forcada I, Vieta E, Mur M. Persistence of cognitive impairment and its negative impact on psychosocial functioning in lithium-treated, euthymic bipolar patients: A 6-year follow-up study. Psychol Med [Internet]. 2013;43(6):1187–96. [Accessed 23 Oct 2023]. Available from: https://www.cambridge.org/core/journals/psychological-medicine/article/persistence-of-cognitive-impairment-and-its-negative-impact-on-psychosocial-functioning-in-lithiumtreated-euthymic-bipolar-patients-a-6year-followup-study/0059C3A1C236EF3E65A9F794CE403636. 22935452 10.1017/S0033291712001948

[23] Mora E, Portella MJ, Forcada I, Vieta E, Mur M. A preliminary longitudinal study on the cognitive and functional outcome of bipolar excellent lithium responders. Compr Psychiatry. 2016;71:25–32. 27592139 10.1016/j.comppsych.2016.07.008

[24] Macoveanu J, Damgaard V, Ysbæk-Nielsen AT, Frangou S, Yatham LN, Chakrabarty T, et al. Early longitudinal changes in brain structure and cognitive functioning in remitted patients with recently diagnosed bipolar disorder. J Affect Disord [Internet]. 2023;339:153–61. [Accessed 21 Sep 2023]. Available from: https://linkinghub.elsevier.com/retrieve/pii/S0165032723008595. 37442440 10.1016/j.jad.2023.07.026

[25] Samamé C, Martino DJ, Strejilevich SA. Longitudinal course of cognitive deficits in bipolar disorder: A meta-analytic study. J Affect Disord [Internet]. 2014;164:130–8. [Accessed 1 Mar 2024]. Available from: https://www.sciencedirect.com/science/article/pii/S0165032714002122. 24856566 10.1016/j.jad.2014.04.028

[26] Samamé C, Cattaneo BL, Richaud MC, Strejilevich S, Aprahamian I. The long-term course of cognition in bipolar disorder: A systematic review and meta-analysis of patient-control differences in test-score changes. Psychol Med. 2022;52(2):217–28. 34763735 10.1017/S0033291721004517

[27] Bora E, Özerdem A. Meta-analysis of longitudinal studies of cognition in bipolar disorder: Comparison with healthy controls and schizophrenia. Psychol Med [Internet]. 2017;47(16):2753–66. [Accessed 10 Oct 2024]. Available from: https://www.cambridge.org/core/journals/psychological-medicine/article/metaanalysis-of-longitudinal-studies-of-cognition-in-bipolar-disorder-comparison-with-healthy-controls-and-schizophrenia/F5C0F14001537531453A7B01BC8D0DE0. 28585513 10.1017/S0033291717001490

[28] Samamé C. Progressive cognitive impairment in bipolar disorder: An assumption that holds true no matter what. Bipolar Disord [Internet]. 2023;25(1):82–82. [Accessed 10 Oct 2024]. Available from: https://onlinelibrary.wiley.com/doi/abs/10.1111/bdi.13289. 36591644 10.1111/bdi.13289

[29] Samamé C. What do psychiatrists do with hypotheses proven false? The case of neuroprogression in bipolar disorders. Psychol Med [Internet]. 2024;54(1):41–2. [Accessed 1 Oct 2024]. Available from: https://www.cambridge.org/core/product/identifier/S0033291723003318/type/journal_article. 37947198 10.1017/S0033291723003318

[30] Strejilevich SA, Samamé C, Quiroz D. “The neuroprogression hypothesis in bipolar disorders: Time for apologies?” Bipolar Disord [Internet]. 2023;25(5):353–4. [Accessed 8 Oct 2024]. Available from: https://onlinelibrary.wiley.com/doi/10.1111/bdi.13358. 37578831 10.1111/bdi.13358

[31] Czepielewski LS, Massuda R, Goi P, Sulzbach-Vianna M, Reckziegel R, Costanzi M, et al. Verbal episodic memory along the course of schizophrenia and bipolar disorder: a new perspective. Eur Neuropsychopharmacol. 2015;25(2):169–75. 25311898 10.1016/j.euroneuro.2014.09.006

[32] Martino DJ, Samamé C, Marengo E, Igoa A, Strejilevich SA. A critical overview of the clinical evidence supporting the concept of neuroprogression in bipolar disorder. Psychiatry Res. 2016;235:1–6. 26723135 10.1016/j.psychres.2015.12.012

[33] López-Jaramillo C, Lopera-Vásquez J, Gallo A, Ospina-Duque J, Bell V, Torrent C, et al. Effects of recurrence on the cognitive performance of patients with bipolar I disorder: Implications for relapse prevention and treatment adherence. Bipolar Disord [Internet]. 2010;12(5):557–67. [Accessed 18 Dec 2023]. Available from: https://onlinelibrary.wiley.com/doi/abs/10.1111/j.1399-5618.2010.00835.x. 20712758 10.1111/j.1399-5618.2010.00835.x

[34] Robinson LJ, Ferrier IN. Evolution of cognitive impairment in bipolar disorder: A systematic review of cross-sectional evidence. Bipolar Disord. 2006;8(2):103–16. 16542180 10.1111/j.1399-5618.2006.00277.x

[35] Torres IJ, DeFreitas VG, DeFreitas CM, Kauer-Sant’Anna M, Bond DJ, Honer WG, et al. Neurocognitive functioning in patients with bipolar I disorder recently recovered from a first manic episode. J Clin Psychiatry. 2010;71(9):1234–42. 20361907 10.4088/JCP.08m04997yel

[36] Yatham LN, Schaffer A, Kessing LV, Miskowiak K, Kapczinski F, Vieta E, et al. Early intervention, relapse prevention, and neuroprogression in bipolar disorder: The evidence matters. Bipolar Disord [Internet]. 2024;26(4):313–6. Available from: https://onlinelibrary.wiley.com/doi/abs/10.1111/bdi.13435. 38664598 10.1111/bdi.13435

[37] Vieta E. Neuroprogression in bipolar disorder: Why right is wrong. Psychol Med [Internet]. 2024:1–3. [Accessed 9 Jul 2024]. Available from: https://www.cambridge.org/core/product/identifier/S0033291724001016/type/journal_article. 10.1017/S0033291724001016 38725354

[38] Thiel K, Meinert S, Winter A, Lemke H, Waltemate L, Breuer F, et al. Reduced fractional anisotropy in bipolar disorder v. major depressive disorder independent of current symptoms. Psychol Med. 2023;53(10):4592–602. 35833369 10.1017/S0033291722001490 PMC10388324

[39] Favre P, Pauling M, Stout J, Hozer F, Sarrazin S, Abé C, et al. Widespread white matter microstructural abnormalities in bipolar disorder: evidence from mega- and meta-analyses across 3033 individuals. Neuropsychopharmacology [Internet]. 2019;44(13):2285–93. [Accessed 21 Nov 2024]. Available from: https://www.ncbi.nlm.nih.gov/pmc/articles/PMC6898371/. 31434102 10.1038/s41386-019-0485-6 PMC6898371

[40] Thiel K, Lemke H, Winter A, Flinkenflügel K, Waltemate L, Bonnekoh L, et al. White and gray matter alterations in bipolar I and bipolar II disorder subtypes compared with healthy controls – exploring associations with disease course and polygenic risk. Neuropsychopharmacology [Internet]. 2024;49(5):814–23. [Accessed 29 Nov 2024]. Available from: https://www.ncbi.nlm.nih.gov/pmc/articles/PMC10948847/. 38332015 10.1038/s41386-024-01812-7 PMC10948847

[41] Holleran L, Kelly S, Alloza C, Agartz I, Andreassen OA, Arango C, et al. The relationship between white matter microstructure and general cognitive ability in patients with schizophrenia and healthy participants in the ENIGMA consortium. AJP [Internet]. 2020;177(6):537–47. [Accessed 30 Aug 2023]. Available from: https://ajp.psychiatryonline.org/doi/10.1176/appi.ajp.2019.19030225. 10.1176/appi.ajp.2019.19030225 PMC7938666 32212855

[42] Meinert S, Nowack N, Grotegerd D, Repple J, Winter NR, Abheiden I, et al. Association of brain white matter microstructure with cognitive performance in major depressive disorder and healthy controls: A diffusion-tensor imaging study. Mol Psychiatry. 2022;27(2):1103–10. 34697453 10.1038/s41380-021-01330-8 PMC9054669

[43] Tanrıkulu AB, İnanlı İ, Arslan S, Çalışkan AM, Çiçek İE, Eren İ. White matter characteristics in the early and late stages of bipolar disorder: A diffusion tensor imaging study. J Affect Disord [Internet]. 2022;308:353–9. [Accessed 2 Nov 2023]. Available from: https://linkinghub.elsevier.com/retrieve/pii/S0165032722003354. 35398113 10.1016/j.jad.2022.04.002

[44] Achalia R, Raju VB, Jacob A, Nahar A, Achalia G, Nagendra B, et al. Comparison of first-episode and multiple-episode bipolar disorder: a surface-based morphometry study. Psychiat Res Neuroimag [Internet]. 2020;302:111110. [Accessed 15 Dec 2023]. Available from: https://linkinghub.elsevier.com/retrieve/pii/S0925492720300822. 10.1016/j.pscychresns.2020.111110 32505904

[45] Nehra R, Chakrabarti S, Pradhan BK, Khehra N. Comparison of cognitive functions between first- and multi-episode bipolar affective disorders. J Affect Disord [Internet]. 2006;93(1–3):185–92. [Accessed 18 Dec 2024]. Available from: https://linkinghub.elsevier.com/retrieve/pii/S016503270600139X. 16678909 10.1016/j.jad.2006.03.013

[46] Rosa A, González-Ortega I, González-Pinto A, Echeburúa E, Comes M, Martínez-Àran A, et al. One-year psychosocial functioning in patients in the early vs. late stage of bipolar disorder. Acta Psychiat Scandin [Internet]. 2012;125(4):335–41. [Accessed 3 Nov 2023]. Available from: https://onlinelibrary.wiley.com/doi/abs/10.1111/j.1600-0447.2011.01830.x. 10.1111/j.1600-0447.2011.01830.x 22283440

[47] Huang KL, Chen MH, Hsu JW, Tsai SJ, Bai YM. Comparison of executive dysfunction, proinflammatory cytokines, and appetite hormones between first-episode and multiple-episode bipolar disorders. CNS Spectr [Internet]. 2023;28(3):351–6. [Accessed 18 Dec 2023]. Available from: https://www.cambridge.org/core/product/identifier/S1092852922000761/type/journal_article. 10.1017/S1092852922000761 35485725

[48] Tremain H, Fletcher K, Murray G. Number of episodes in bipolar disorder: the case for more thoughtful conceptualization and measurement. Bipolar Disorders [Internet]. 2020;22(3):231–44. [Accessed 18 Dec 2023]. Available from: https://onlinelibrary.wiley.com/doi/abs/10.1111/bdi.12872. 31730294 10.1111/bdi.12872

[49] Tremain H, Fletcher K, Scott J, McEnery C, Berk M, Murray G. The influence of stage of illness on functional outcomes after psychological treatment in bipolar disorder: A systematic review. Bipolar Disorders [Internet]. 2020;22(7):666–92. [Accessed 1 Oct 2024]. Available from: https://onlinelibrary.wiley.com/doi/abs/10.1111/bdi.12974 32621794 10.1111/bdi.12974

[50] Kapczinski F, Magalhães PVS, Balanzá-Martinez V, Dias VV, Frangou S, Gama CS, et al. Staging systems in bipolar disorder: an international society for bipolar disorders task force report. Acta Psychiat Scandin [Internet]. 2014;130(5):354–63. [Accessed 3 Nov 2024]. Available from: https://onlinelibrary.wiley.com/doi/abs/10.1111/acps.12305. 10.1111/acps.12305 24961757

[51] Malhi GS, Bell E, Morris G, Hamilton A. Staging bipolar disorder: An alluring proposition. Bipolar Disorders [Internet]. 2020;22(7):660–3. [Accessed 29 Nov 2024]. Available from: https://onlinelibrary.wiley.com/doi/abs/10.1111/bdi.13020. 33063437 10.1111/bdi.13020

[52] Bauer M, Andreassen OA, Geddes JR, Vedel Kessing L, Lewitzka U, Schulze TG, et al. Areas of uncertainties and unmet needs in bipolar disorders: Clinical and research perspectives. Lancet Psychiatry. 2018;5(11):930–9. 30146246 10.1016/S2215-0366(18)30253-0

[53] Kircher T, Wöhr M, Nenadic I, Schwarting R, Schratt G, Alferink J, et al. Neurobiology of the major psychoses: A translational perspective on brain structure and function-the FOR2107 consortium. Eur Arch Psychiatry Clin Neurosci. 2019;269(8): 949–62. 30267149 10.1007/s00406-018-0943-x

[54] Wittchen HU, Wunderlich U, Gruschwitz S, Zaudig M. Strukturiertes Klinisches interview Für DSM-IV. Achse I: Psychische Störungen. In: Interviewheft Und Beurteilungsheft. Eine Deutschsprachige, Erweiterte Bearbeitung Der Amerikanischen Originalversion Des SKID I. Goettingen: Hogrefe; 1997.

[55] McGuffin P, Farmer A, Harvey I. A polydiagnostic application of operational criteria in studies of psychotic illness. Development and reliability of the OPCRIT system. Arch Gen Psychiatry. 1991;48(8):764–70. 1883262 10.1001/archpsyc.1991.01810320088015

[56] Hamilton M. A rating scale for depression. J Neurol Neurosurg Psychiatry. 1960;23(1):56–62. 14399272 10.1136/jnnp.23.1.56 PMC495331

[57] Young RC, Biggs JT, Ziegler VE, Meyer DA. A rating scale for mania: Reliability, validity and sensitivity. Br J Psychiatry. 1978;133:429–35. 728692 10.1192/bjp.133.5.429

[58] Saß H, Wittchen HU, Zaudig M, Houben I. Diagnostisches und Statistisches Manual Psychischer Störungen: Textrevision – DSM-IV-TR. Göttingen; Bern, Toronto; Seattle: Hogrefe; Verlag für Psychologie; 2003.

[59] Hassel S, Almeida JR, Kerr N, Nau S, Ladouceur CD, Fissell K, et al. Elevated striatal and decreased dorsolateral prefrontal cortical activity in response to emotional stimuli in euthymic bipolar disorder: no associations with psychotropic medication load. Bipolar Disord. 2008;10(8):916–27. 19594507 10.1111/j.1399-5618.2008.00641.x PMC2711546

[60] Vogelbacher C, Möbius TWD, Sommer J, Schuster V, Dannlowski U, Kircher T, et al. The Marburg-Münster affective disorders cohort study (MACS): a quality assurance protocol for MR neuroimaging data. NeuroImage. 2018;172(December 2017): 450–60. 29410079 10.1016/j.neuroimage.2018.01.079

[61] Jenkinson M, Beckmann CF, Behrens TEJ, Woolrich MW, Smith SM. FSL. NeuroImage [Internet]. 2012;62(2):782–90. [Accessed 25 Oct 2024]. Available from: https://www.sciencedirect.com/science/article/pii/S1053811911010603. 21979382 10.1016/j.neuroimage.2011.09.015

[62] Smith SM, Jenkinson M, Woolrich MW, Beckmann CF, Behrens TEJ, Johansen-Berg H, et al. Advances in functional and structural MR image analysis and implementation as FSL. NeuroImage [Internet]. 2004;23:S208–19. Available from: https://www.sciencedirect.com/science/article/pii/S1053811904003933. 15501092 10.1016/j.neuroimage.2004.07.051

[63] Woolrich MW, Jbabdi S, Patenaude B, Chappell M, Makni S, Behrens T, et al. Bayesian analysis of neuroimaging data in FSL. NeuroImage. 2009;45(1 Suppl):S173–186. 19059349 10.1016/j.neuroimage.2008.10.055

[64] Feldman HM, Yeatman JD, Lee ES, Barde LHF, Gaman-Bean S. Diffusion tensor imaging: A review for pediatric researchers and clinicians. J Dev Behav Pediatr. 2010;31(4):346–56. 20453582 10.1097/DBP.0b013e3181dcaa8b PMC4245082

[65] Alexander AL, Lee JE, Lazar M, Field AS. Diffusion tensor imaging of the brain. Neurotherapeutics [Internet]. 2007;4(3):316–29. [Accessed 11 Jan 2025]. Available from: https://www.sciencedirect.com/science/article/pii/S1878747923006530. 17599699 10.1016/j.nurt.2007.05.011 PMC2041910

[66] Smith SM, Jenkinson M, Johansen-Berg H, Rueckert D, Nichols TE, Mackay CE, et al. Tract-based spatial statistics: Voxelwise analysis of multi-subject diffusion data. NeuroImage. 2006;31(4):1487–505. 16624579 10.1016/j.neuroimage.2006.02.024

[67] Winkler AM, Ridgway GR, Webster MA, Smith SM, Nichols TE. Permutation inference for the general linear model. NeuroImage [Internet]. 2014;92:381–97. [Accessed 25 Oct 2024]. Available from: https://www.sciencedirect.com/science/article/pii/S1053811914000913. 24530839 10.1016/j.neuroimage.2014.01.060 PMC4010955

[68] Smith SM, Nichols TE. Threshold-free cluster enhancement: addressing problems of smoothing, threshold dependence and localisation in cluster inference. NeuroImage. 2009;44(1):83–98. 18501637 10.1016/j.neuroimage.2008.03.061

[69] Benjamini Y, Hochberg Y. Controlling the false discovery rate: A practical and powerful approach to multiple testing. J R Stat Soc Ser B (Methodol). 1995;57(1):289–300.

[70] Lavagnino L, Cao B, Mwangi B, Wu MJ, Sanches M, Zunta-Soares GB, et al. Changes in the corpus callosum in women with late-stage bipolar disorder. Acta Psychiatr Scand. 2015;131(6):458–64. 25640667 10.1111/acps.12397 PMC4932908

[71] Ferro A, Bonivento C, Delvecchio G, Bellani M, Perlini C, Dusi N, et al. Longitudinal investigation of the parietal lobe anatomy in bipolar disorder and its association with general functioning. Psychiatr Res Neuroimaging. 2017;267:22–31. 10.1016/j.pscychresns.2017.06.010 28732208

[72] Forcada I, Papachristou E, Mur M, Christodoulou T, Jogia J, Reichenberg A, et al. The impact of general intellectual ability and white matter volume on the functional outcome of patients with bipolar disorder and their relatives. J Affect Disord [Internet]. 2011;130(3):413–20. [Accessed 23 Oct 2024]. Available from: https://www.sciencedirect.com/science/article/pii/S0165032710006774. 21112093 10.1016/j.jad.2010.10.048

[73] Bonnín CM, Jiménez E, Solé B, Torrent C, Radua J, Reinares M, et al. Lifetime psychotic symptoms, subthreshold depression and cognitive impairment as barriers to functional recovery in patients with bipolar disorder. J Clin Med [Internet]. 2019;8(7):1046. [Accessed 7 Aug 2023]. Available from: https://www.mdpi.com/2077-0383/8/7/1046. 31323795 10.3390/jcm8071046 PMC6679346

[74] Sanchez-Moreno J, Martinez-Aran A, Tabarés-Seisdedos R, Torrent C, Vieta E, Ayuso-Mateos JL. Functioning and disability in bipolar disorder: An extensive review. Psychother Psychosom [Internet]. 2009;78(5):285–97. [Accessed 7 Aug 2023]. Available from: https://www.karger.com/Article/FullText/228249. 19602917 10.1159/000228249

[75] Sanchez-Moreno J, Bonnin CM, González-Pinto A, Amann BL, Solé B, Balanzá-Martinez V, et al. Factors associated with poor functional outcome in bipolar disorder: Sociodemographic, clinical, and neurocognitive variables. Acta Psychiatr Scand [Internet]. 2018;138(2):145–54. [Accessed 10 Oct 2024]. Available from: https://onlinelibrary.wiley.com/doi/abs/10.1111/acps.12894. 29726004 10.1111/acps.12894

[76] Burdick KE, Millett CE, Yocum AK, Altimus CM, Andreassen OA, Aubin V, et al. Predictors of functional impairment in bipolar disorder: Results from 13 cohorts from seven countries by the global bipolar cohort collaborative. Bipolar Disorders [Internet]. 2022;24(7):709–19. [Accessed 7 Aug 2023]. Available from: https://onlinelibrary.wiley.com/doi/abs/10.1111/bdi.13208. 35322518 10.1111/bdi.13208 PMC9500115

[77] Solé B, Bonnin CM, Jiménez E, Torrent C, Torres I, Varo C, et al. Heterogeneity of functional outcomes in patients with bipolar disorder: A cluster-analytic approach. Acta Psychiatrica Scandinavica [Internet]. 2018 [cited 2023 Jul 12];137(6):516–27. [Accessed 12 Jul 2023]. Available from: https://onlinelibrary.wiley.com/doi/abs/10.1111/acps.12871. 29508379 10.1111/acps.12871

[78] Léda-Rêgo G, Bezerra-Filho S, Miranda-Scippa Â. Functioning in euthymic patients with bipolar disorder: a systematic review and meta-analysis using the functioning assessment short test. Bipolar Disord. 2020;22(6):569–81. 32243046 10.1111/bdi.12904

[79] Caruana GF, Carruthers SP, Berk M, Rossell SL, Van Rheenen TE. To what extent does white matter map to cognition in bipolar disorder? A systematic review of the evidence. Prog Neuro-Psychopharmacol Biol Psychiatry. 2024;128. 10.1016/j.pnpbp.2023.110868 37797735

[80] Duarte JA, Massuda R, Goi PD, Vianna-Sulzbach M, Colombo R, Kapczinski F, et al. White matter volume is decreased in bipolar disorder at early and late stages. Trends Psychiatry Psychother. 2018;40(4):277–84. 30570099 10.1590/2237-6089-2017-0025

[81] Librenza-Garcia D, Suh J, Watts D, Ballester P, Minuzzi L, Kapczinski F, et al. Structural and functional brain correlates of neuroprogression in bipolar disorder. In: Young AH, Juruena MF, editors. Bipolar disorder: from neuroscience to treatment current topics in Behavioral neurosciences. Cham: Springer; 2020. 10.1007/7854_2020_177 33040317

[82] Weathers J, Lippard ETC, Spencer L, Pittman B, Wang F, Blumberg HP. Longitudinal diffusion tensor imaging study of adolescents and uoung adults with bipolar disorder. J Am Acad Child Adoles Psychiatr [Internet]. 2018;57(2): 111–7 [cited 4 Oct 2024]. Available from: https://www.sciencedirect.com/science/article/pii/S0890856717318646. 10.1016/j.jaac.2017.11.014 PMC5806147 29413143

[83] Moorhead TWJ, McKirdy J, Sussmann JED, Hall J, Lawrie SM, Johnstone EC, et al. Progressive gray matter loss in patients with bipolar disorder. Biol Psychiatry 2007;62(8):894–900. 17617385 10.1016/j.biopsych.2007.03.005

[84] Hafeman DM, Chang KD, Garrett AS, Sanders EM, Phillips ML. Effects of medication on neuroimaging findings in bipolar disorder: An updated review. Bipolar Disord [Internet]. 2012;14(4):375–410 [Accessed 11 Jan 2025]. Available from: https://onlinelibrary.wiley.com/doi/abs/10.1111/j.1399-5618.2012.01023.x. 22631621 10.1111/j.1399-5618.2012.01023.x

[85] Canales-Rodríguez EJ, Verdolini N, Alonso-Lana S, Torres ML, Panicalli F, Argila-Plaza I, et al. Widespread intra-axonal signal fraction abnormalities in bipolar disorder from multicompartment diffusion MRI: Sensitivity to diagnosis, association with clinical features and pharmacologic treatment. Hum Brain Map [Internet]. 2023;44(12):4605–22 [Accessed 21 Sep 2023]. Available from: https://onlinelibrary.wiley.com/doi/abs/10.1002/hbm.26405. 10.1002/hbm.26405 PMC10365239 37357976

[86] Hozer F, Houenou J. Can neuroimaging disentangle bipolar disorder? J Affect Disord [Internet]. 2016;195:199–214 [Accessed 10 Dec 2024]. Available from: https://www.sciencedirect.com/science/article/pii/S0165032715307539. 26896814 10.1016/j.jad.2016.01.039

[87] Chen YL, Tu PC, Huang TH, Bai YM, Su TP, Chen MH, et al. Identifying subtypes of bipolar disorder based on clinical and neurobiological characteristics. Sci Rep [Internet] 2021; 11:17082 [Accessed 16 Dec 2024]. Available from: https://www.nature.com/articles/s41598-021-96645-5 34429498 10.1038/s41598-021-96645-5 PMC8385023

[88] Argyropoulos GD, Christidi F, Karavasilis E, Bede P, Antoniou A, Velonakis G, et al. Predominant polarity as a neurobiological specifier in bipolar disorder: evidence from a multimodal neuroimaging study. Prog Neuro-Psychopharmacol Biol Psychiatry. 2023;123:110718. 10.1016/j.pnpbp.2023.110718 36634808

[89] Altamura AC, Maggioni E, Dhanoa T, Ciappolino V, Paoli RA, Cremaschi L, et al. The impact of psychosis on brain anatomy in bipolar disorder: A structural MRI study. J Affect Disord. 2018;233:100–9. 29223329 10.1016/j.jad.2017.11.092

[90] Sarrazin S, d’Albis MA, McDonald C, Linke J, Wessa M, Phillips M, et al. Corpus callosum area in patients with bipolar disorder with and without psychotic features: An international multicentre study. J Psychiatry Neurosci. 2015;40(5):352–9. 26151452 10.1503/jpn.140262 PMC4543098

[91] Sweet JA, Gao K, Chen Z, Tatsuoka C, Calabrese JR, Sajatovic M, et al. Cingulum bundle connectivity in treatment-refractory compared to treatment-responsive patients with bipolar disorder and healthy controls: A tractography and surgical targeting analysis. J Neurosurg. 2022;137(3):709–21. 35061996 10.3171/2021.11.JNS211833 PMC10193487

[92] Belizario GO, Gigante AD, de Almeida Rocca CC, Lafer B. Cognitive impairments and predominant polarity in bipolar disorder: A cross-sectional study. Int J Bipolar Disord 2017;5:15. 28332122 10.1186/s40345-017-0085-5 PMC5427058

[93] Abé C, Ekman CJ, Sellgren C, Petrovic P, Ingvar M, Landén M. Manic episodes are related to changes in frontal cortex: A longitudinal neuroimaging study of bipolar disorder 1. Brain. 2015;138(11):3440–8. 26373602 10.1093/brain/awv266

[94] Aminoff SR, Onyeka IN, Ødegaard M, Simonsen C, Lagerberg TV, Andreassen OA, et al. Lifetime and point prevalence of psychotic symptoms in adults with bipolar disorders: A systematic review and meta-analysis. Psychol Med [Internet]. 2022;52(13):2413–25 [Accessed 21 Jan 2025]. Available from: https://www.cambridge.org/core/journals/psychological-medicine/article/lifetime-and-point-prevalence-of-psychotic-symptoms-in-adults-with-bipolar-disorders-a-systematic-review-and-metaanalysis/31492842FCD4D49E1FDE86B332F062AA. 36016504 10.1017/S003329172200201X PMC9647517

[95] Sehmbi M, Rowley CD, Minuzzi L, Kapczinski F, Kwiecien JM, Bock NA, et al. Age-related deficits in intracortical myelination in young adults with bipolar disorder type I. J Psychiatr Neurosci [Internet] 2019; 44(2): 79–88 [Accessed 21 Jan 2025]. Available from: https://www.jpn.ca/content/44/2/79. 10.1503/jpn.170220 PMC6397039 30525334

[96] Preti MG, Baglio F, Laganà MM, Griffanti L, Nemni R, Clerici M, et al. Assessing corpus callosum changes in Alzheimer’s disease: Comparison between tract-based spatial statistics and atlas-based tractography. PLoS One. 2012;7(4):e35856. 22545143 10.1371/journal.pone.0035856 PMC3335803

