# Supplementary material for: Disease progression in bipolar disorder in relation to white matter microstructure: A comprehensive approach based on staging models
Source: Eur Psychiatry. 2025 Sep 15;68(1):e148. doi: 10.1192/j.eurpsy.2025.10105 (PMC12538178; doi:10.1192/j.eurpsy.2025.10105)
Supplement: Thiel et al. supplementary material [file S0924933825101053sup001.docx]

**Supplementary Material**

**Supplement 1. Medication Load Index**

Current psychopharmacological medication intake was assessed through a previously used composite score, the medication load index (1). Each psychopharmacological agent was assigned a value between 0 and 2 depending on whether that medication was absent (=0), the dose was equal to or lower than the average dose (low=1) or the dose was higher than average (high=2). The average dose was defined by the average daily dose range recommended by the Physician’s-Desk-Reference. By calculating the sum of all medication scores per participant, the resulting score reflected both the number and daily dose of all current psychopharmacological medications taken by the participant.

**Supplement 2. DTI acquisition, quality assurance, preprocessing and analysis**

**DTI data acquisition**

Acquisition of DTI data was done using two 3T whole body MR scanners (Marburg: Tim Trio, Siemens, Erlangen, Germany; Münster: Prisma fit, Siemens, Erlangen, Germany). All images were quality controlled according to the respective protocol of the MACS study (2). Pulse sequence parameters were standardised across both scanning sites to the extent allowed by each platform to maximise the signal-to-noise ratio. Fifty-six axial slices with no gap were measured with an isotropic voxel size of 2.5x2.5x2.5mm³ (TE=90ms, TS=7300ms), using a GRAPPA acceleration factor of 2. Five non-diffusion-weighted (DW) images (b=0s/mm2) and 2x30 DW images with a b-value of 1000s/mm² were acquired. For quality assurance of the data, the open-source software DTIPrep (3) was used with default options. In case of artifacts, individual images of a given participant were eliminated, and a participant was excluded from further analyses if more than 20% of images were affected. During the study, participants lay on their backs with their heads in a supine position, supported by additional padding to reduce motion artifacts.

**DTI data preprocessing**

Preprocessing was implemented in FSL6.0.1 (http://fsl.fmrib.ox.ac.uk/fsl/fslwiki/, FMRIB, Oxford Center for Functional MRI of the Brain, University of Oxford, Department of Clinical Neurology, John Radcliffe Hospital, Oxford, United Kingdom) (4–6). The DW images were corrected for head motion and eddy current induced distortions using “eddy” from FSL (7), and b-vectors were rotated accordingly. After removal of non-brain tissue using the Brain Extraction Tool (BET) in FSL (8), the first b0 image from each participant was used as reference for alignment. A diffusion tensor was estimated for each voxel, using ‘DTIFIT’ within FMRIB’s Diffusion Toolbox (FDT) (9) followed by the generation of tensor-derived maps. Fractional anisotropy (FA), mean diffusivity (MD), radial diffusivity (RD) and axial diffusivity (AD) were estimated for each voxel per participant. FA measure the directionality of water diffusion on a scale from 0 (indicating isotropic diffusion) to 1 (indicating completely anisotropic diffusion). It is calculated as the normalized variance of the three eigenvalues about their mean. MD represents average water diffusion and is calculated as the mean of the three eigenvalues. AD and RD are specific measures of diffusivity parallel and perpendicular to the principal direction of the axonal fibres, respectively. AD is equivalent to the first eigenvalue, whereas RD is calculated as the average of the second and third eigenvalues (10). The values should be interpreted cautiously, since the number of fibers, fiber crossings and general fiber orientation can also affect diffusion metrics in healthy fiber structure (11). Nevertheless, increased MD and decreased FA are indicative of neuronal injury, while increased RD reflects demyelination and decreased AD signifies axonal damage (12).

**DTI data preprocessing**

Using FMRIB’s non-linear registration tool, all FA images were aligned to the FMRIB58 FA template brain in 1×1×1 mm³ Montreal Neurological Institute (MNI) standard space. All registered FA images were averaged and a threshold of 0.2 was applied to create a WM skeleton representing the centers of the tracts common to all participants. Each participant’s aligned FA data were then projected onto the mean skeleton mask by searching for maximum FA values perpendicular to the local skeleton direction.

**DTI data analysis**

Cluster size and MNI coordinates of the peak voxel of all significant clusters were obtained using the “cluster” tool in FSL. Anatomical labels of significant clusters, based on the “JHU White-Matter Labels” atlas (19), were identified with FSL’s „atlasquery“ command. For plots created in R, using „fslstats“ in FSL, mean FA from voxels forming significant clusters were extracted per participant. Effect sizes were calculated based on the mean t-value of all significant voxels provided by FSL (using „fslstats“) and respective sample sizes (20).

All analyses were repeated for MD, AD, and RD. The total intracranial volume (TIV) was extracted from T1 images using the Computational Anatomy Toolbox (CAT12, http://www.neuro.uni-jena.de/cat, v933). The T1 sequence has been described elsewhere (2).

**Supplement 3. Effects in RD, MD and AD**

**Analysis 1. WM microstructural differences between HC, BD_first_ and BD_multiple_**

There were significant main effects of group in RD (*F*-contrast: *p*_tfce-FWE_=.001, total *k*=10221 voxels in three clusters, *Table S4*) and MD (*F*-contrast: *p*_tfce-FWE_=.006, total *k*=8311 voxels in four clusters, *Table S4*). These were further examined by pairwise comparisons which revealed significantly higher RD/MD values in BD_multiple_ compared with HC (RD: *p*_tfce-FWE_=.001, *k*=36396 voxels in five clusters, MD: *p*_tfce-FWE_=.006, *k*=20768 voxels in three clusters) as well as compared with BD_first_ (RD: *p*_tfce-FWE=_.008, *k*=24250 voxels in three clusters, MD: *p*_tfce-FWE=_.035, *k*=6986 voxels in twelve clusters). In contrast, BD_first_ patients did not show significantly different RD/MD values compared with HC (RD: *p*_tfce-FWE_=.611, MD: *p*_tfce-FWE_=.348). The effects were mainly localized in the corpus callosum, the corona radiata, and the superior longitudinal fasciculus (*Table S5*). After FDR correction for multiple testing, significance of the results for RD remained unchanged (BD_multiple_ > HC: *p*_tfce-FWE_ =.003, BD_first_ > HC: *p*_tfce-FWE_ =.012). For MD, only the comparison BD_multiple_ > HC was found to be significant (*p*_tfce-FWE_=.018), whereas the comparison BD_first_ > HC narrowly missed significance (*p*_tfce-FWE_=.053).

The difference between the two BD groups remained significant even when adjusting for additional clinical characteristics like acute depressive and manic symptomatology, age of onset, comorbidities, or medication intake (*Table S4*).

For AD, the main effect of group was not significant (*p*_tfce-FWE_=.743).

**Analysis 2. WM microstructural differences between HC, BD_rem_ and BD_chron_**

When categorizing BD patients based on their previous remission quality, the *F*-contrast revealed a significant main effect of group in RD (*p*_tfce-FWE_=.022, total *k*=1420 voxels in ten clusters, *Table S4*). No significant main effect emerged for MD (*p*_tfce-FWE_=.089) and AD *p*_tfce-FWE_=.108). Pairwise post-hoc *t*-contrasts revealed significantly lower RD values for HC compared with BD_chron_ (*p*_tfce-FWE_=.005, one cluster with k=50593 voxels)_,_ as well as BD_rem_, albeit less pronounced (*p*_tfce-FWE_=.038, total k=1174 voxels in three clusters). BD_rem_ and BD_chron_ however, did not show significant differences (*p*_tfce-FWE_=.207). Differences between HC and BD_rem_ were mainly localized in the corpus callousum and the superior corona radiata, whereas the comparison between HC and BD_chron_ also included the anterior and posterior corona radiata, internal and external capsule, posterior thalamic radiation and superior longitudinal fasciculus (*Table S5*).

After FDR correction for multiple testing, only the comparison BD_chron_ > HC was found to be significant (*p*_tfce-FWE_ =.015). In the comparison BD_rem_ > HC, the significance threshold was narrowly missed (*p*_tfce-FWE_ =.057). As the difference between the BD groups was not significant, no further analyses including correction for clinical characteristics were conducted.

**Analysis 3.** **Association between GAF scores and WM microstructure in remitted BD patients**

The linear regression analysis investigating an association between the GAF score and diffusion metrics in euthymic BD patients yielded a significant negative association in RD (*p_tfce-FWE_*=.002, four clusters with total k=42285 voxels) as well as MD (*p_tfce-FWE_*=.006, one cluster with k=32350 voxels) (*Table S4*). Neither a positive (*p_tfce-FWE_*=.484) nor a negative (*p_tfce-FWE_*=.345) association was found for AD.

**Supplement 4. Analysis 3 (association between GAF scores and WM microstructure)**

**conducted in the whole BD sample**

For this analysis, *n*=2 BD patients had to be excluded due to missing values of the GAF score (resulting in *n*=151 BD patients). The linear regression analysis investigating an association between the GAF score and FA in all BD patients yielded a significant positive association (*p_tfce-FWE_*=.038, 16 cluster with k=6720 voxels). For RD, MD and AD, no significant association was found (RD: *p_tfce-FWE_*=.074, MD: *p_tfce-FWE_*=.172, AD: *p_tfce-FWE_*=.70).

**Supplement 5: Supplementary Tables**

Table S1. Demographic and clinical characteristics of BD patients in (partial) remission.

|  | **BD patients in (partial) remission**  **(n=75)** |
| --- | --- |
| **Demographics** |  |
| Age | 40.8 ± 11.7 |
| Biological Sex (f/m) | 43/32 |
| Site (Marburg/Münster) | 30/45 |
| **Questionnaires** |  |
| HDRS scores | 5.04 ± 5.08 |
| YMRS scores | 2.13 ± 3.62 |
| GAF scores | 68.4 ± 11.3 |
| **Psychiatric Medication** |  |
| Medication load index | 2.53 ± 1.78 |
| None (yes/no) | 14/61 |
| Antidepressants (yes/no) | 32/43 |
| Antipsychotics (yes/no) | 38/37 |
| Anticonvulsives (yes/no) | 21/54 |
| Lithium (yes/no) | 25/50 |
| **Clinical characteristics** |  |
| Bipolar subtype (BD1/BD2) | 44/31 |
| Remission status (partly-/fully remitted) | 36/39 |
| Age of Onset | 23.0 ± 9.9 |
| Lifetime psychiatric comorbidity (yes/no) | 33/42 |
| Number of depressive episodes | 6.59 ± 5.90 (n=74) |
| Duration of depressive episodes (months) | 48.7 ± 82.5 (n=65) |
| Number of (hypo-)manic episodes | 3.99 ± 3.82 |
| Duration of (hypo-)manic episodes (months) | 8.19 ± 10.2 (n=65) |
| Number of inpatient treatments | 3.57 ± 3.55 (n=72) |
| Duration of inpatient treatments (weeks) | 31.4 ± 32.5 (n=71) |
| Duration of sick leave (months) | 15.9 ± 23.3 (n=68) |
| Duration of retirement (months) | 20.5 ± 54.2 (n=55) |
| Note: Data are mean ± SD or frequencies. BD=bipolar disorder, HDRS=21-item Hamilton Depression Rating Scale, GAF= General Assessment of Functioning Scale, YMRS=Young Mania Rating Scale. | |

Table S2. Cluster sizes and MNI coordinates of the peak voxel of all significant clusters, derived with the “cluster” tool implemented in FSL.

| Analysis 1: HC vs. BD_first_ vs. BD_multiple_ | | | | | |
| --- | --- | --- | --- | --- | --- |
| Main effect of group (F-Test) | | | | | |
| Cluster | k | *p_tfce-FWE_* | x | y | z |
| FA |  |  |  |  |  |
| 7 | 6319 | .001 | -3 | 23 | 14 |
| 6 | 324 | .027 | -30 | -68 | 1 |
| 5 | 160 | .015 | -42 | -29 | 31 |
| 4 | 23 | .044 | 23 | -47 | 55 |
| 3 | 15 | .047 | 22 | -41 | 48 |
| 2 | 1 | .05 | -19 | -55 | 26 |
| 1 | 1 | .05 | 21 | -45 | 47 |
| RD |  |  |  |  |  |
| 3 | 8938 | .001 | 18 | 6 | 36 |
| 2 | 1268 | .008 | -41 | -32 | 31 |
| 1 | 15 | .048 | -15 | 6 | 51 |
| MD |  |  |  |  |  |
| 4 | 7926 | .006 | 28 | -44 | 24 |
| 3 | 230 | .037 | 25 | -3 | 34 |
| 2 | 127 | .047 | 32 | -24 | 40 |
| 1 | 28 | .047 | 27 | -29 | 40 |
| AD |  | .743 |  |  |  |
| Post-hoc t-test: HC vs. BD_first_ | | | | | |
| FA: HC > BD_first_ |  | .688 |  |  |  |
| RD: HC < BD_first_ |  | .611 |  |  |  |
| MD: HC < BD_first_ |  | .348 |  |  |  |
| Post-hoc t-test: HC vs. BD_multiple_ | | | | | |
| Cluster | k | *p_tfce-FWE_* | x | y | z |
| FA: HC > BD_multiple_ |  |  |  |  |  |
| 1 | 45480 | <.001 | -4 | 26 | 11 |
| RD: HC < BD_multiple_ |  |  |  |  |  |
| 5 | 35501 | .001 | -3 | 25 | 12 |
| 4 | 709 | .046 | -11 | -24 | 1 |
| 3 | 173 | .046 | 27 | -31 | -2 |
| 2 | 8 | .05 | 16 | 9 | -16 |
| 1 | 5 | .05 | 20 | -33 | 2 |
| MD: HC < BD_multiple_ |  |  |  |  |  |
| 3 | 20287 | .005 | 5 | 25 | 12 |
| 2 | 473 | .044 | -23 | -16 | 8 |
| 1 | 8 | .05 | -11 | -24 | 1 |
| Post-hoc t-test: BD_first_ vs. BD_multiple_ | | | | | |
| Cluster | k | *p_tfce-FWE_* | x | y | z |
| FA: BD_first_ > BD_multiple_ | | | | | |
| 7 | 22140 | .003 | 18 | 30 | 24 |
| 6 | 1035 | .044 | -44 | -39 | 4 |
| 5 | 217 | .046 | -48 | -19 | -23 |
| 4 | 74 | .047 | -37 | -1 | -29 |
| 3 | 7 | .05 | -53 | -47 | -1 |
| 2 | 3 | .05 | -44 | -5 | -23 |
| 1 | 2 | .05 | -43 | -1 | -24 |
| FA: BD_first_ > BD_multiple,_ controlled for medication load index | | | | | |
| 3 | 24253 | .003 | 19 | 31 | 17 |
| 2 | 67 | .047 | 37 | -2 | -15 |
| 1 | 51 | .049 | 42 | -10 | -23 |
| FA: BD_first_ > BD_multiple,_ controlled for lithium (yes/no) | | | | | |
| 11 | 26319 | .003 | 18 | 30 | 24 |
| 10 | 432 | .042 | 28 | 1 | 45 |
| 9 | 130 | .047 | -26 | -31 | -4 |
| 8 | 78 | .05 | -32 | 28 | 23 |
| 7 | 70 | .05 | -32 | 37 | 20 |
| 6 | 66 | .047 | 36 | -56 | 9 |
| 5 | 47 | .049 | -27 | 24 | 33 |
| 4 | 10 | .05 | -31 | 20 | 28 |
| 3 | 3 | .05 | -31 | 37 | 24 |
| 2 | 2 | .05 | -31 | 34 | 33 |
| 1 | 2 | .05 | -29 | 27 | 34 |
| FA: BD_first_ > BD_multiple,_ controlled for antidepressants (yes/no) | | | | | |
| 1 | 25565 | .003 | 18 | 31 | 22 |
| FA: BD_first_ > BD_multiple,_ controlled for anticonvulsives (yes/no) | | | | | |
| 4 | 14114 | .01 | 18 | 30 | 23 |
| 3 | 532 | .044 | 15 | -14 | 58 |
| 2 | 61 | .048 | 10 | -12 | 33 |
| 1 | 30 | .05 | 10 | -59 | 26 |
| FA: BD_first_ > BD_multiple,_ controlled for antipsychotics (yes/no) | | | | | |
| 10 | 21064 | .004 | 19 | 31 | 17 |
| 9 | 312 | .046 | -43 | -38 | 5 |
| 8 | 113 | .048 | -44 | -10 | -26 |
| 7 | 93 | .047 | -41 | -40 | -9 |
| 6 | 78 | .046 | -29 | -82 | -4 |
| 5 | 45 | .047 | -38 | -1 | -29 |
| 4 | 24 | .049 | -30 | -78 | -10 |
| 3 | 15 | .049 | -36 | -80 | 2 |
| 2 | 3 | .05 | -53 | -47 | -1 |
| 1 | 2 | .05 | -21 | -86 | -7 |
| FA: BD_first_ > BD_multiple,_ controlled for acute manic symptoms (YMRS score) | | | | | |
| 1 | 21856 | .003 | 18 | 30 | 24 |
| FA: BD_first_ > BD_multiple,_ controlled for acute depressive symptoms (HDRS score) | | | | | |
| 5 | 22126 | .003 | 18 | 34 | 12 |
| 4 | 1133 | .044 | -42 | -35 | 2 |
| 3 | 434 | .046 | -44 | -10 | -26 |
| 2 | 77 | .048 | -53 | -47 | -1 |
| 1 | 66 | .049 | -55 | -27 | -13 |
| FA: BD_first_ > BD_multiple,_ controlled for number of inpatient treatments | | | | | |
| 23 | 29516 | .002 | 18 | 34 | 12 |
| 22 | 3059 | .034 | -44 | -11 | -26 |
| 21 | 2106 | .033 | 42 | -10 | -23 |
| 20 | 387 | .039 | 43 | -25 | 1 |
| 19 | 347 | .046 | 8 | -24 | 11 |
| 18 | 105 | .046 | -5 | -17 | 15 |
| 17 | 88 | .047 | 0 | 5 | 7 |
| 16 | 47 | .046 | 36 | -56 | 10 |
| 15 | 43 | .049 | 36 | -34 | -22 |
| 14 | 27 | .049 | 3 | -14 | 0 |
| 13 | 18 | .049 | 30 | -37 | -20 |
| 12 | 14 | .049 | -17 | -21 | -8 |
| 11 | 12 | .047 | 8 | -1 | 36 |
| 10 | 12 | .049 | 55 | -46 | -6 |
| 9 | 9 | .049 | -22 | -24 | 4 |
| 8 | 9 | .05 | 41 | -30 | -15 |
| 7 | 7 | .05 | 39 | -44 | -10 |
| 6 | 6 | .049 | 38 | -26 | -24 |
| 5 | 6 | .049 | 10 | -12 | 33 |
| 4 | 3 | .05 | 4 | -1 | 5 |
| 3 | 2 | .05 | 40 | -40 | -9 |
| 2 | 2 | .05 | 9 | -2 | 33 |
| 1 | 1 | .05 | -14 | -15 | 20 |
| FA: BD_first_ > BD_multiple,_ controlled for age-squared | | | | | |
| 1 | 24211 | .003 | 18 | 30 | 23 |
| FA: BD_first_ > BD_multiple,_ controlled for lifetime comorbidity (yes/no) | | | | | |
| 16 | 27526 | .002 | 18 | 30 | 23 |
| 15 | 516 | .04 | 28 | 1 | 45 |
| 14 | 426 | .046 | 46 | 4 | 19 |
| 13 | 243 | .046 | -17 | -21 | -8 |
| 12 | 213 | .045 | -26 | -31 | -4 |
| 11 | 154 | .048 | -19 | 20 | -2 |
| 10 | 105 | .048 | -22 | 19 | -10 |
| 9 | 86 | .048 | -27 | -19 | 55 |
| 8 | 61 | .049 | -21 | -31 | 11 |
| 7 | 11 | .05 | 17 | -55 | 49 |
| 6 | 7 | .05 | 55 | -16 | 24 |
| 5 | 4 | .05 | -22 | 24 | -3 |
| 4 | 3 | .05 | -41 | -28 | 2 |
| 3 | 2 | .05 | 19 | -49 | 30 |
| 2 | 1 | .05 | -22 | -32 | -4 |
| 1 | 1 | .05 | 19 | -51 | 30 |
| FA: BD_first_ > BD_multiple,_ controlled for age of onset | | | | | |
| 3 | 26257 | .005 | 18 | 31 | 22 |
| 2 | 159 | .048 | 36 | -3 | -16 |
| 1 | 25 | .049 | 31 | -21 | 2 |
| FA: BD_first_ > BD_multiple,_ controlled for BD subtype | | | | | |
| 1 | 19577 | .007 | 18 | 29 | 27 |
| RD: BD_first_ < BD_multiple_ | | | | | |
| 3 | 24118 | .008 | 20 | 31 | 19 |
| 2 | 116 | .049 | -36 | -8 | 38 |
| 1 | 16 | .05 | -37 | -8 | 46 |
| RD: BD_first_ < BD_multiple,_ controlled for medication load index | | | | | |
| 1 | 17689 | .011 | 20 | 31 | 19 |
| RD: BD_first_ < BD_multiple,_ controlled for lithium (yes/no) | | | | | |
| 1 | 28823 | .005 | 20 | 31 | 19 |
| RD: BD_first_ < BD_multiple,_ controlled for antidepressants (yes/no) | | | | | |
| 1 | 26011 | .007 | 18 | 14 | 30 |
| RD: BD_first_ < BD_multiple,_ controlled for anticonvulsives (yes/no) | | | | | |
| 3 | 8415 | .02 | 19 | -2 | 41 |
| 2 | 196 | .048 | -33 | -39 | 34 |
| 1 | 66 | .045 | -42 | -29 | 31 |
| RD: BD_first_ < BD_multiple,_ controlled for antipsychotics (yes/no) | | | | | |
| 1 | 26105 | .007 | 20 | 34 | 17 |
| RD: BD_first_ < BD_multiple,_ controlled for acute manic symptoms (YMRS score) | | | | | |
| 11 | 22305 | .007 | 17 | 14 | 33 |
| 10 | 120 | .048 | -49 | -24 | -20 |
| 9 | 118 | .048 | -52 | -32 | 7 |
| 8 | 114 | .048 | -37 | -1 | -29 |
| 7 | 92 | .048 | -53 | -46 | -2 |
| 6 | 87 | .049 | -44 | -39 | -7 |
| 5 | 30 | .05 | -44 | -11 | -26 |
| 4 | 30 | .05 | -56 | -30 | -11 |
| 3 | 22 | .05 | -44 | -29 | -1 |
| 2 | 8 | .05 | -54 | -27 | -13 |
| 1 | 3 | .05 | -36 | -22 | -4 |
| RD: BD_first_ < BD_multiple,_ controlled for acute depressive symptoms (HDRS score) | | | | | |
| 1 | 24158 | .008 | 20 | 31 | 19 |
| RD: BD_first_ < BD_multiple,_ controlled for number of inpatient treatments | | | | | |
| 1 | 29111 | .006 | 19 | 29 | 21 |
| RD: BD_first_ < BD_multiple,_ controlled for age-squared | | | | | |
| 7 | 23685 | .007 | 2 | 25 | 12 |
| 6 | 365 | .049 | -43 | -35 | 2 |
| 5 | 123 | .049 | -53 | -47 | -1 |
| 4 | 118 | .049 | -48 | -19 | -23 |
| 3 | 89 | .049 | -54 | -27 | -13 |
| 2 | 17 | .05 | -37 | -1 | -29 |
| 1 | 10 | .05 | -47 | -21 | -3 |
| RD: BD_first_ < BD_multiple,_ controlled for lifetime comorbidity (yes/no) | | | | | |
| 4 | 28841 | .006 | 20 | 31 | 19 |
| 3 | 399 | .046 | -27 | 24 | 33 |
| 2 | 180 | .048 | 46 | 4 | 18 |
| 1 | 21 | .05 | 23 | 18 | -12 |
| RD: BD_first_ < BD_multiple,_ controlled for age of onset | | | | | |
| 1 | 25245 | .008 | 18 | 15 | 31 |
| RD: BD_first_ < BD_multiple,_ controlled for BD subtype | | | | | |
| 1 | 17282 | .013 | 19 | 29 | 25 |
| MD: BD_first_ < BD_multiple_ | | | | | |
| 12 | 3571 | .035 | 18 | -36 | 30 |
| 11 | 2058 | .039 | -21 | -38 | 29 |
| 10 | 991 | .038 | -33 | -54 | 29 |
| 9 | 139 | .049 | 35 | -66 | 22 |
| 8 | 117 | .049 | 34 | -27 | 36 |
| 7 | 39 | .05 | 16 | -30 | 54 |
| 6 | 31 | .05 | 39 | -55 | 31 |
| 5 | 19 | .05 | 32 | -14 | 40 |
| 4 | 10 | .05 | 36 | -55 | 11 |
| 3 | 6 | .05 | 30 | -48 | 35 |
| 2 | 4 | .05 | 36 | -51 | 34 |
| 1 | 1 | .05 | 34 | -48 | 33 |
| MD: BD_first_ < BD_multiple,_ controlled for medication load index | | | | | |
| 6 | 808 | .045 | 18 | -36 | 29 |
| 5 | 413 | .049 | 12 | 19 | 22 |
| 4 | 92 | .049 | 3 | -24 | 23 |
| 3 | 92 | .049 | 22 | -52 | 26 |
| 2 | 3 | .05 | 19 | -6 | 44 |
| 1 | 2 | .05 | 11 | -18 | 29 |
| MD: BD_first_ < BD_multiple,_ controlled for lithium (yes/no) | | | | | |
| 2 | 5679 | .03 | 18 | -36 | 29 |
| 1 | 4075 | .036 | -33 | -52 | 29 |
| MD: BD_first_ < BD_multiple,_ controlled for antidepressants (yes/no) | | | | | |
| 10 | 3998 | .032 | 14 | -36 | 26 |
| 9 | 3502 | .032 | -34 | -54 | 28 |
| 8 | 139 | .048 | 35 | -66 | 22 |
| 7 | 47 | .049 | 38 | -55 | 32 |
| 6 | 46 | .049 | 15 | -27 | 55 |
| 5 | 34 | .049 | 29 | -48 | 36 |
| 4 | 22 | .05 | 19 | 39 | 29 |
| 3 | 21 | .05 | 35 | -53 | 31 |
| 2 | 20 | .049 | 34 | -50 | 34 |
| 1 | 17 | .05 | 36 | -55 | 11 |
| MD: BD_first_ < BD_multiple,_ controlled for anticonvulsives (yes/no) | | | | | |
| 1 | 52426 | ..063 | 18 | -37 | 29 |
| MD: BD_first_ < BD_multiple,_ controlled for antipsychotics (yes/no) | | | | | |
| 7 | 3585 | .037 | 22 | -39 | 28 |
| 6 | 1613 | .043 | -21 | -38 | 29 |
| 5 | 932 | .041 | -33 | -53 | 30 |
| 4 | 198 | .049 | -29 | -26 | 34 |
| 3 | 101 | .049 | 35 | -66 | 22 |
| 2 | 80 | .05 | -36 | -2 | 35 |
| 1 | 1 | .05 | -31 | -25 | 35 |
| MD: BD_first_ < BD_multiple,_ controlled for acute manic symptoms (YMRS score) | | | | | |
| 5 | 3496 | .035 | 18 | -36 | 30 |
| 4 | 1391 | .041 | -21 | -38 | 29 |
| 3 | 913 | .039 | -33 | -54 | 29 |
| 2 | 486 | .046 | -21 | -50 | 14 |
| 1 | 20 | .05 | 34 | -27 | 36 |
| MD: BD_first_ < BD_multiple,_ controlled for acute depressive symptoms (HDRS score) | | | | | |
| 11 | 3973 | .036 | 18 | -36 | 29 |
| 10 | 2052 | .04 | -21 | -37 | 30 |
| 9 | 985 | .04 | -34 | -54 | 28 |
| 8 | 183 | .049 | 39 | -55 | 31 |
| 7 | 175 | .048 | 35 | -66 | 22 |
| 6 | 110 | .049 | 45 | -39 | 31 |
| 5 | 35 | .049 | 35 | -58 | 10 |
| 4 | 6 | .05 | 45 | -29 | 33 |
| 3 | 3 | .05 | 43 | -45 | 34 |
| 2 | 1 | .05 | 44 | -24 | 32 |
| 1 | 1 | .05 | 34 | -44 | 33 |
| MD: BD_first_ < BD_multiple,_ controlled for number of inpatient treatments | | | | | |
| 2 | 5657 | .031 | 16 | -35 | 27 |
| 1 | 3951 | .035 | -33 | -52 | 29 |
| MD: BD_first_ < BD_multiple,_ controlled for age-squared | | | | | |
| 1 | 11910 | .028 | 18 | -36 | 29 |
| MD: BD_first_ < BD_multiple,_ controlled for lifetime comorbidity (yes/no) | | | | | |
| 2 | 5985 | .027 | 25 | -38 | 27 |
| 1 | 4266 | .034 | -33 | -52 | 29 |
| MD: BD_first_ < BD_multiple,_ controlled for age of onset | | | | | |
| 3 | 5487 | .029 | 18 | -36 | 29 |
| 2 | 2504 | .038 | -21 | -38 | 29 |
| 1 | 932 | .043 | -33 | -52 | 29 |
| Analysis 2: HC vs. BD_rem_ vs. BD_chron_ | | | | | |
| Main effect of group (F-Test) | | | | | |
| Cluster | k | *p_tfce-FWE_* | x | y | z |
| FA |  |  |  |  |  |
| 5 | 1505 | .005 | -13 | -15 | 31 |
| 4 | 222 | .028 | 18 | -8 | 49 |
| 3 | 18 | .047 | -3 | 6 | 23 |
| 2 | 17 | .047 | -6 | -3 | 27 |
| 1 | 2 | .049 | -8 | -10 | 28 |
| RD |  |  |  |  |  |
| 10 | 491 | .022 | -12 | -2 | 31 |
| 9 | 379 | .019 | -17 | -31 | 31 |
| 8 | 334 | .031 | 20 | -9 | 43 |
| 7 | 114 | .04 | 13 | 1 | 32 |
| 6 | 67 | .045 | -21 | -24 | 43 |
| 5 | 22 | .047 | -2 | 5 | 24 |
| 4 | 5 | .049 | -7 | -6 | 28 |
| 3 | 4 | .049 | -6 | -24 | 25 |
| 2 | 2 | .05 | -6 | 11 | 23 |
| 1 | 2 | .049 | -8 | -10 | 28 |
| MD |  | .089 |  |  |  |
| AD |  | .108 |  |  |  |
| Post-hoc t-test: HC vs. BD_rem_ | | | | | |
| Cluster | k | *p_tfce-FWE_* | x | y | z |
| FA: HC > BD_rem_ |  |  |  |  |  |
| 4 | 628 | .031 | -12 | 0 | 30 |
| 3 | 564 | .042 | -11 | 17 | 23 |
| 2 | 227 | .046 | 13 | -2 | 32 |
| 1 | 7 | .05 | -13 | -24 | 30 |
| RD: HC < BD_rem_ |  |  |  |  |  |
| 3 | 865 | .038 | -12 | 2 | 30 |
| 2 | 175 | .046 | -4 | 17 | 19 |
| 1 | 134 | .047 | 14 | -1 | 32 |
| Post-hoc t-test: HC vs. BD_chron_ | | | | | |
| Cluster | k | *p_tfce-FWE_* | x | y | z |
| FA: HC > BD_chron_ |  |  |  |  |  |
| 1 | 39297 | <.001 | -2 | 28 | 10 |
| RD: HC < BD_chron_ |  |  |  |  |  |
| 1 | 30593 | .005 | -29 | -45 | 18 |
| Post-hoc t-test: BD_rem_ vs. BD_chron_ | | | | | |
| Cluster | k | *p_tfce-FWE_* | x | y | z |
| FA: BD_rem_ > BD_chron_ |  | .075 |  |  |  |
| RD: BD_rem_ < BD_chron_ |  | .207 |  |  |  |
| Analysis 3: effect of GAF |  |  |  |  |  |
| Cluster | k | *p_tfce-FWE_* | x | y | z |
| FA: positive effect |  |  |  |  |  |
| 1 | 43114 | .001 | 12 | 30 | -9 |
| FA: positive effect, controlled for medication load index | | | | | |
| 1 | 43583 | .002 | 18 | -15 | 52 |
| FA: positive effect, controlled for lithium (yes/no) | | | | | |
| 1 | 40139 | .001 | 12 | 30 | -10 |
| FA: positive effect, controlled for antidepressants (yes/no) | | | | | |
| 1 | 43709 | .001 | 13 | 36 | -14 |
| FA: positive effect, controlled for anticonvulsives (yes/no) | | | | | |
| 4 | 40126 | .002 | 13 | 36 | -14 |
| 3 | 166 | .046 | 56 | -35 | -8 |
| 2 | 20 | .05 | 51 | -37 | -13 |
| 1 | 3 | .05 | 50 | -29 | -21 |
| FA: positive effect, controlled for antipsychotics (yes/no) | | | | | |
| 1 | 35748 | .005 | 16 | 35 | -6 |
| FA: positive effect, controlled for acute manic symptoms (YMRS score) | | | | | |
| 1 | 42836 | .001 | 12 | 31 | -8 |
| FA: positive effect, controlled for acute depressive symptoms (HDRS score) | | | | | |
| 5 | 27387 | .007 | 12 | 30 | -9 |
| 4 | 82 | .048 | -37 | -47 | -12 |
| 3 | 6 | .05 | -47 | -18 | 29 |
| 2 | 6 | .05 | -44 | -19 | 30 |
| 1 | 1 | .05 | -41 | -9 | 26 |
| FA: positive effect, controlled for number of inpatient treatments | | | | | |
| 1 | 44921 | .001 | 8 | 26 | -15 |
| FA: positive effect, controlled for number of manic episodes | | | | | |
| 1 | 36385 | .005 | 18 | -35 | 30 |
| FA: positive effect, controlled for number of depressive episodes | | | | | |
| 1 | 39050 | .007 | 17 | -34 | 29 |
| FA: positive effect, controlled for lifetime comorbidity (yes/no) | | | | | |
| 1 | 43069 | .001 | 12 | 31 | -8 |
| FA: positive effect,_,_ controlled for age of onset | | | | | |
| 1 | 44046 | .001 | 13 | 34 | -13 |
| FA: positive effect,_,_ controlled for BD subtype | | | | | |
| 1 | 40656 | .002 | 13 | 34 | -13 |
| FA: negative effect |  | .89 |  |  |  |
| RD: positive effect |  | .718 |  |  |  |
| RD: negative effect |  |  |  |  |  |
| 4 | 42207 | .002 | 17 | 26 | -14 |
| 3 | 55 | .05 | 19 | -10 | 2 |
| 2 | 22 | .049 | 27 | 38 | 9 |
| 1 | 1 | .05 | 27 | 39 | 14 |
| RD: negative effect, controlled for medication load index | | | | | |
| 3 | 41085 | .002 | 15 | 39 | -13 |
| 2 | 62 | .049 | 49 | -52 | 11 |
| 1 | 5 | .05 | 38 | -78 | 1 |
| RD: negative effect, controlled for lithium (yes/no) | | | | | |
| 1 | 41141 | .002 | 7 | 18 | -18 |
| RD: negative effect, controlled for antidepressants (yes/no) | | | | | |
| 1 | 42026 | .002 | 16 | 39 | -10 |
| RD: negative effect, controlled for anticonvulsives (yes/no) | | | | | |
| 1 | 41491 | .003 | 22 | -20 | 37 |
| RD: negative effect, controlled for antipsychotics (yes/no) | | | | | |
| 5 | 35359 | .004 | 16 | 43 | -12 |
| 4 | 68 | .049 | 57 | -34 | -8 |
| 3 | 31 | .05 | 14 | 52 | 26 |
| 2 | 2 | .05 | 53 | -37 | -7 |
| 1 | 1 | .05 | 39 | -6 | -22 |
| RD: negative effect,_,_ controlled for acute manic symptoms (YMRS score) | | | | | |
| 1 | 42522 | .002 | 31 | 41 | -3 |
| RD: negative effect,_,_ controlled for acute depressive symptoms (HDRS score) | | | | | |
| 1 | 35901 | .004 | 16 | 43 | -12 |
| RD: negative effect,_,_ controlled for number of inpatient treatments | | | | | |
| 4 | 44043 | .001 | 16 | 39 | -9 |
| 3 | 17 | .049 | -29 | 13 | -1 |
| 2 | 4 | .05 | -29 | -9 | 16 |
| 1 | 1 | .05 | -29 | 14 | -5 |
| RD: negative effect,_,_ controlled for number of manic episodes | | | | | |
| 6 | 36686 | .004 | 23 | 29 | 19 |
| 5 | 604 | .046 | 48 | -46 | -1 |
| 4 | 27 | .05 | -19 | 21 | -4 |
| 3 | 24 | .05 | -26 | 17 | 38 |
| 2 | 7 | .05 | -41 | -2 | -28 |
| 1 | 1 | .05 | -12 | -93 | 16 |
| RD: negative effect,_,_ controlled for number of depressive episodes | | | | | |
| 2 | 38678 | .002 | 22 | 36 | -1 |
| 1 | 14 | .05 | 48 | -46 | -1 |
| RD: negative effect,_,_ controlled for lifetime comorbidity (yes/no) | | | | | |
| 1 | 42199 | .002 | 31 | 41 | -3 |
| RD: negative effect,_,_ controlled for age of onset | | | | | |
| 1 | 41750 | .002 | 23 | 29 | 19 |
| RD: negative effect,_,_ controlled for BD subtype | | | | | |
| 1 | 40650 | .003 | 32 | 42 | -3 |
| MD: positive effect |  | .641 |  |  |  |
| MD: negative effect |  |  |  |  |  |
| 1 | 32350 | .006 | 24 | 31 | 15 |
| MD: negative effect, controlled for medication load index | | | | | |
| 1 | 34624 | .006 | -14 | 33 | -8 |
| MD: negative effect, controlled for lithium (yes/no) | | | | | |
| 1 | 31077 | .007 | 22 | 34 | 12 |
| MD: negative effect, controlled for antidepressants (yes/no) | | | | | |
| 1 | 32157 | .006 | 22 | 34 | 12 |
| MD: negative effect, controlled for anticonvulsives (yes/no) | | | | | |
| 1 | 33324 | .006 | 23 | 35 | 8 |
| MD: negative effect, controlled for antipsychotics (yes/no) | | | | | |
| 1 | 25660 | .011 | 27 | 12 | 25 |
| MD: negative effect,_,_ controlled for acute manic symptoms (YMRS score) | | | | | |
| 1 | 33051 | .006 | 22 | 34 | 12 |
| MD: negative effect,_,_ controlled for acute depressive symptoms (HDRS score) | | | | | |
| 1 | 28487 | .009 | -33 | -35 | 35 |
| MD: negative effect,_,_ controlled for number of inpatient treatments | | | | | |
| 1 | 34212 | .005 | 26 | 13 | 30 |
| MD: negative effect,_,_ controlled for number of manic episodes | | | | | |
| 4 | 29740 | .009 | 28 | 28 | 19 |
| 3 | 42 | .049 | 7 | -32 | 22 |
| 2 | 11 | .05 | 19 | 12 | 7 |
| 1 | 1 | .05 | 10 | -35 | 22 |
| MD: negative effect,_,_ controlled for number of depressive episodes | | | | | |
| 1 | 30283 | .007 | 26 | 12 | 30 |
| MD: negative effect,_,_ controlled for lifetime comorbidity (yes/no) | | | | | |
| 1 | 32345 | .006 | 24 | 31 | 15 |
| MD: negative effect,_,_ controlled for age of onset | | | | | |
| 1 | 31030 | .006 | 21 | 36 | -6 |
| MD: negative effect,_,_ controlled for BD subtype | | | | | |
| 1 | 29115 | .007 | 26 | 12 | 30 |
| AD: positive effect |  | .484 |  |  |  |
| AD: negative effect |  | .345 |  |  |  |
|  |  |  |  |  |  |
| Analysis 3: effect of GAF in the whole BD sample (*n*=151) |  |  |  |  |  |
| Cluster | k | *p_tfce-FWE_* | x | y | z |
| FA: positive effect |  |  |  |  |  |
| 16 | 2555 | .041 | -14 | 33 | -8 |
| 15 | 1046 | .038 | 27 | -25 | -1 |
| 14 | 876 | .044 | 19 | 33 | 24 |
| 13 | 640 | .047 | 21 | -4 | 18 |
| 12 | 543 | .042 | 19 | -36 | 30 |
| 11 | 327 | .043 | -42 | -13 | -15 |
| 10 | 281 | .048 | -20 | -14 | -4 |
| 9 | 207 | .045 | 17 | -15 | 55 |
| 8 | 75 | .048 | -20 | -23 | -1 |
| 7 | 57 | .048 | 17 | 10 | 51 |
| 6 | 46 | .048 | 16 | 1 | 52 |
| 5 | 20 | .05 | -37 | -4 | -28 |
| 4 | 13 | .05 | -25 | -33 | 0 |
| 3 | 12 | .05 | 35 | -45 | 8 |
| 2 | 12 | .05 | 0 | -35 | 20 |
| 1 | 10 | .05 | 29 | -42 | 25 |
| FA: negative effect |  | .807 |  |  |  |
| RD: positive effect |  | .879 |  |  |  |
| RD: negative effect |  | .074 |  |  |  |
| MD: positive effect |  | .811 |  |  |  |
| MD: negative effect |  | .172 |  |  |  |
| AD: positive effect |  | .30 |  |  |  |
| AD: negative effect |  | .837 |  |  |  |
| Note AD=axial diffusivity, BD=participants with bipolar disorder, FA=fractional anisotropy, HDRS=Hamilton Depression Rating Scale, HC=healthy controls, k=voxel count, MD=mean diffusivity, MNI=Montreal Neurological Institute, RD=radial diffusivity, YMRS=Young Mania Rating Scale. | | | | | |

Table S3. Anatomical regions comprising the significant effects of the analyses based on the “JHU ICBM-DTI-81 White-Matter Labels”, as implemented in FSL (19). The numbers represent the average probability of the mask of the significant cluster to be a member of the different labeled regions within the atlas, calculated with the FSL tool “atlasquery”.

| Analysis | Contrast | Region | Hemisphere | Percentage |
| --- | --- | --- | --- | --- |
| Analysis 1: HC vs. BD_first_ vs. BD_multiple_ | FA: Main effect of group (F-Test) | Genu of corpus callosum |  | 13.7255 |
|  |  | Body of corpus callosum |  | 35.7555 |
|  |  | Splenium of corpus callosum |  | 5.9977 |
|  |  | Anterior corona radiata | R | 6.5744 |
|  |  | Anterior corona radiata | L | 1.6148 |
|  |  | Superior corona radiata | R | 4.0369 |
|  |  | Superior corona radiata | L | 2.7682 |
|  |  | Posterior corona radiata | R | 1.0381 |
|  |  | Posterior corona radiata | L | 1.9608 |
|  |  | Posterior thalamic radiation (include optic radiation) | L | 3.1142 |
|  |  | Superior longitudinal fasciculus | L | 1.1534 |
|  | RD: Main effect of group (F-Test) | Genu of corpus callosum |  | 7.6336 |
|  |  | Body of corpus callosum |  | 23.3588 |
|  |  | Splenium of corpus callosum |  | 5.6489 |
|  |  | Retrolenticular part of internal capsule | R | 0.0763 |
|  |  | Anterior corona radiata | R | 2.9771 |
|  |  | Anterior corona radiata | L | 2.0611 |
|  |  | Superior corona radiata | R | 3.4351 |
|  |  | Superior corona radiata | L | 3.8931 |
|  |  | Posterior corona radiata | R | 4.3511 |
|  |  | Posterior corona radiata | L | 3.8931 |
|  |  | Posterior thalamic radiation (include optic radiation) | R | 0.5344 |
|  |  | Posterior thalamic radiation (include optic radiation) | L | 2.4427 |
|  |  | Cingulum (cingulate gyrus) | R | 0.0763 |
|  |  | Cingulum (cingulate gyrus) | L | 0.3053 |
|  |  | Superior longitudinal fasciculus | L | 3.0534 |
|  |  | Tapetum | R | 0.1527 |
|  | MD: Main effect of group (F-Test) | Genu of corpus callosum |  | 3.7072 |
|  |  | Body of corpus callosum |  | 20.057 |
|  |  | Splenium of corpus callosum |  | 4.943 |
|  |  | Anterior limb of internal capsule | L | 0.1901 |
|  |  | Posterior limb of internal capsule | L | 3.0418 |
|  |  | Retrolenticular part of internal capsule | L | 0.0951 |
|  |  | Anterior corona radiata | R | 2.5665 |
|  |  | Anterior corona radiata | L | 0.8555 |
|  |  | Superior corona radiata | R | 4.4677 |
|  |  | Superior corona radiate | L | 9.6008 |
|  |  | Posterior corona radiata | R | 4.943 |
|  |  | Posterior corona radiata | L | 4.3726 |
|  |  | Posterior thalamic radiation (include optic radiation) | L | 0.3802 |
|  |  | Superior longitudinal fasciculus | R | 0.5703 |
|  |  | Superior Longitudinal fasciculus | L | 3.0418 |
|  |  | Tapetum | R | 0.1901 |
|  | FA: HC > BD_multiple_ | Genu of corpus callosum |  | 3.3733 |
|  |  | Body of corpus callosum |  | 6.8336 |
|  |  | Splenium of corpus callosum |  | 3.4081 |
|  |  | Fornix (column and body of fornix) |  | 0.3478 |
|  |  | Superior cerebellar peduncle | L | 0.0869 |
|  |  | Cerebral peduncle | R | 0.313 |
|  |  | Cerebral peduncle | L | 0.4173 |
|  |  | Anterior limb of internal capsule | L | 0.8694 |
|  |  | Posterior limb of internal capsule | R | 0.2087 |
|  |  | Posterior limb of internal capsule | L | 0.5912 |
|  |  | Retrolenticular part of internal capsule | R | 0.7303 |
|  |  | Retrolenticular part of internal capsule | L | 0.7303 |
|  |  | Anterior corona radiata | R | 2.1561 |
|  |  | Anterior corona radiata | L | 2.1388 |
|  |  | Superior corona radiata | R | 1.3737 |
|  |  | Superior corona radiata | L | 1.6867 |
|  |  | Posterior corona radiata | R | 1.3737 |
|  |  | Posterior corona radiata | L | 1.1998 |
|  |  | Posterior thalamic radiation (include optic radiation) | R | 1.7214 |
|  |  | Posterior thalamic radiation (include optic radiation) | L | 1.6693 |
|  |  | Sagittal stratum (include inferior longitidinal fasciculus and inferior fronto-occipital fasciculus) | R | 0.6434 |
|  |  | Sagittal stratum (include inferior longitidinal fasciculus and inferior fronto-occipital fasciculus) | L | 0.6434 |
|  |  | External capsule | R | 1.7041 |
|  |  | External capsule | L | 2.2083 |
|  |  | Cingulum (cingulate gyrus) |  | 0.1217 |
|  |  | Cingulum (cingulate gyrus) | L | 0.8868 |
|  |  | Fornix (cres) / Stria terminalis (can not be resolved with current resolution) | R | 0.3478 |
|  |  | Fornix (cres) / Stria terminalis (can not be resolved with current resolution) | L | 0.4173 |
|  |  | Superior longitudinal fasciculus | R | 1.9997 |
|  |  | Superior longitudinal fasciculus | L | 1.9127 |
|  |  | Uncinate fasciculus | R | 0.1565 |
|  |  | Uncinate fasciculus | L | 0.1913 |
|  |  | Tapetum | R | 0.0522 |
|  |  | Tapetum | L | 0.0174 |
|  | RD: HC < BD_multiple_ | Genu of corpus callosum |  | 3.6838 |
|  |  | Body of corpus callosum |  | 8.3369 |
|  |  | Splenium of corpus callosum |  | 3.3175 |
|  |  | Fornix (column and body of fornix) |  | 0.4524 |
|  |  | Cerebral peduncle | R | 0.0215 |
|  |  | Cerebral peduncle | L | 0.3878 |
|  |  | Anterior limb of internal capsule | L | 0.237 |
|  |  | Posterior limb of internal capsule | R | 0.3447 |
|  |  | Posterior limb of internal capsule | L | 0.7324 |
|  |  | Retrolenticular part of internal capsule | R | 0.7755 |
|  |  | Retrolenticular part of internal capsule | L | 0.9263 |
|  |  | Anterior corona radiata | R | 2.4558 |
|  |  | Anterior corona radiata | L | 2.7143 |
|  |  | Superior corona radiata | R | 1.9173 |
|  |  | Superior corona radiata | L | 2.6497 |
|  |  | Posterior corona radiata | R | 1.7449 |
|  |  | Posterior corona radiata | L | 1.5941 |
|  |  | Posterior thalamic radiation (include optic radiation) | R | 2.1112 |
|  |  | Posterior thalamic radiation (include optic radiation) | L | 1.9388 |
|  |  | Sagittal stratum (include inferior longitidinal fasciculus and inferior fronto-occipital fasciculus) | R | 0.7755 |
|  |  | Sagittal stratum (include inferior longitidinal fasciculus and inferior fronto-occipital fasciculus) | L | 0.6032 |
|  |  | External capsule |  | 0.9694 |
|  |  | External capsule | L | 1.9604 |
|  |  | Cingulum (cingulate gyrus) | R | 0.6894 |
|  |  | Cingulum (cingulate gyrus) | L | 1.034 |
|  |  | Fornix (cres) / Stria terminalis (can not be resolved with current resolution) | R | 0.3016 |
|  |  | Fornix (cres) / Stria terminalis (can not be resolved with current resolution) | L | 0.4524 |
|  |  | Superior longitudinal fasciculus | R | 2.305 |
|  |  | Superior longitudinal fasciculus | L | 2.6282 |
|  |  | Uncinate fasciculus | R | 0.1293 |
|  |  | Uncinate fasciculus | L | 0.2154 |
|  |  | Tapetum | R | 0.0431 |
|  | MD: HC < BD_multiple_ | Genu of corpus callosum |  | 3.0916 |
|  |  | Body of corpus callosum |  | 11.9466 |
|  |  | Splenium of corpus callosum |  | 4.2748 |
|  |  | Cerebral peduncle | L | 0.4198 |
|  |  | Anterior limb of internal capsule | L | 0.1527 |
|  |  | Posterior limb of internal capsule | L | 1.7176 |
|  |  | Retrolenticular part of internal capsule | R | 0.458 |
|  |  | Retrolenticular part of internal capsule | L | 0.3435 |
|  |  | Anterior corona radiata | R | 2.7481 |
|  |  | Anterior corona radiata | L | 3.5878 |
|  |  | Superior corona radiata | R | 2.8244 |
|  |  | Superior corona radiata | L | 4.771 |
|  |  | Posterior corona radiata | R | 2.7481 |
|  |  | Posterior corona radiata | L | 2.4809 |
|  |  | Posterior thalamic radiation (include optic radiation) | R | 1.9847 |
|  |  | Posterior thalamic radiation (include optic radiation) | L | 0.7634 |
|  |  | External capsule | L | 0.1908 |
|  |  | Cingulum (cingulate gyrus) | R | 0.1908 |
|  |  | Cingulum (cingulate gyrus) | L | 0.1145 |
|  |  | Superior longitudinal fasciculus | R | 3.5878 |
|  |  | Superior longitudinal fasciculus | L | 4.313 |
|  |  | Tapetum | R | 0.0763 |
|  | FA: BD_first_ > BD_multiple_ | Genu of corpus callosum |  | 4.6933 |
|  |  | Body of corpus callosum |  | 11.1633 |
|  |  | Splenium of corpus callosum |  | 4.1569 |
|  |  | Anterior limb of internal capsule | R | 0.1341 |
|  |  | Anterior limb of internal capsule | L | 0.2682 |
|  |  | Posterior limb of internal capsule | L | 0.067 |
|  |  | Retrolenticular part of internal capsule | R | 0.1341 |
|  |  | Retrolenticular part of internal capsule | L | 0.5028 |
|  |  | Anterior corona radiata | R | 4.5592 |
|  |  | Anterior corona radiata | L | 4.0898 |
|  |  | Superior corona radiata | R | 1.2068 |
|  |  | Superior corona radiata | L | 2.6819 |
|  |  | Posterior corona radiata | R | 2.0114 |
|  |  | Posterior corona radiata | L | 1.2739 |
|  |  | Posterior thalamic radiation (include optic radiation) | R | 0.5364 |
|  |  | Posterior thalamic radiation (include optic radiation) | L | 1.6091 |
|  |  | Sagittal stratum (include inferior longitidinal fasciculus and inferior fronto-occipital fasciculus) | L | 0.6034 |
|  |  | External capsule | R | 0.0335 |
|  |  | External capsule | L | 1.1733 |
|  |  | Cingulum (cingulate gyrus) | R | 0.5699 |
|  |  | Cingulum (cingulate gyrus) | L | 0.6705 |
|  |  | Fornix (cres) / Stria terminalis (can not be resolved with current resolution) | L | 0.1006 |
|  |  | Superior longitudinal fasciculus | R | 1.8103 |
|  |  | Superior longitudinal fasciculus | L | 3.3188 |
|  |  | Superior fronto-occipital fasciculus (could be a part of anterior internal capsule) | R | 0.4693 |
|  |  | Superior fronto-occipital fasciculus (could be a part of anterior internal capsule) | L | 0.1341 |
|  |  | Tapetum |  | 0.067 |
|  |  | Tapetum | L | 0.0335 |
|  | RD: BD_first_ < BD_multiple_ | Genu of corpus callosum |  | 3.308 |
|  |  | Body of corpus callosum |  | 9.7916 |
|  |  | Splenium of corpus callosum |  | 3.4072 |
|  |  | Anterior limb of internal capsule | L | 0.7278 |
|  |  | Retrolenticular part of internal capsule | R | 0.0662 |
|  |  | Retrolenticular part of internal capsule | L | 0.6285 |
|  |  | Anterior corona radiata | R | 3.2749 |
|  |  | Anterior corona radiata | L | 2.8118 |
|  |  | Superior corona radiata | R | 1.1909 |
|  |  | Superior corona radiata | L | 2.2825 |
|  |  | Posterior corona radiata | R | 2.1833 |
|  |  | Posterior corona radiata | L | 1.5878 |
|  |  | Posterior thalamic radiation (include optic radiation) | R | 1.3893 |
|  |  | Posterior thalamic radiation (include optic radiation) | L | 1.5547 |
|  |  | Sagittal stratum (include inferior longitidinal fasciculus and inferior fronto-occipital fasciculus) | R | 0.0662 |
|  |  | Sagittal stratum (include inferior longitidinal fasciculus and inferior fronto-occipital fasciculus) | L | 0.6616 |
|  |  | External capsule | L | 0.397 |
|  |  | Cingulum (cingulate gyrus) | R | 0.8601 |
|  |  | Cingulum (cingulate gyrus) | L | 0.4631 |
|  |  | Fornix (cres) / Stria terminalis (can not be resolved with current resolution) | L | 0.0992 |
|  |  | Superior longitudinal fasciculus | R | 1.2901 |
|  |  | Superior longitudinal fasciculus | L | 2.6795 |
|  |  | Uncinate fasciculus | L | 0.0662 |
|  |  | Tapetum | R | 0.0331 |
|  | MD: BD_first_ < BD_multiple_ | Genu of corpus callosum |  | 3.4843 |
|  |  | Body of corpus callosum |  | 13.3566 |
|  |  | Splenium of corpus callosum |  | 6.2718 |
|  |  | Anterior corona radiata | R | 3.3682 |
|  |  | Superior corona radiata | R | 3.1359 |
|  |  | Superior corona radiata | L | 0.3484 |
|  |  | Posterior corona radiata | R | 5.5749 |
|  |  | Posterior corona radiata | L | 3.0197 |
|  |  | Posterior thalamic radiation (include optic radiation) | R | 0.2323 |
|  |  | Posterior thalamic radiation (include optic radiation) | L | 0.6969 |
|  |  | Superior longitudinal fasciculus | R | 0.5807 |
|  |  | Superior longitudinal fasciculus | L | 2.5552 |
| Analysis 2: HC vs. BD_rem_ vs. BD_chron_ | FA: Main effect of group (F-Test) | Body of corpus callosum |  | 36.6379 |
|  |  | Splenium of corpus callosum |  | 8.1897 |
|  |  | Superior corona radiata | L | 9.4828 |
|  |  | Posterior corona radiata | R | 1.7241 |
|  |  | Posterior corona radiata | L | 7.7586 |
|  |  | Posterior thalamic radiation (include optic radiation) | L | 1.7241 |
|  | RD: Main effect of group (F-Test) | Body of corpus callosum |  | 29.6703 |
|  |  | Splenium of corpus callosum |  | 3.2967 |
|  |  | Superior corona radiata | R | 7.6923 |
|  |  | Superior corona radiata | L | 12.0879 |
|  |  | Posterior corona radiata | L | 10.989 |
|  | FA: HC > BD_rem_ | Genu of corpus callosum |  | 1.7442 |
|  |  | Body of corpus callosum |  | 79.0698 |
|  |  | Splenium of corpus callosum |  | 5.814 |
|  |  | Anterior corona radiata | R | 2.3256 |
|  |  | Superior corona radiata | R | 2.3256 |
|  |  | Superior corona radiata | L | 1.1628 |
|  |  | Posterior corona radiata | L | 1.1628 |
|  | RD: HC < BD_rem_ | Genu of corpus callosum |  | 10.9677 |
|  |  | Body of corpus callosum |  | 80.6452 |
|  |  | Superior corona radiata | R | 4.5161 |
|  |  | Superior corona radiata | L | 3.871 |
|  | FA: HC > BD_chron_ | Pontine crossing tract (a part of MCP) |  | 0.0203 |
|  |  | Genu of corpus callosum |  | 3.1389 |
|  |  | Body of corpus callosum |  | 6.5209 |
|  |  | Splenium of corpus callosum |  | 2.4706 |
|  |  | Corticospinal tract | R | 0.2228 |
|  |  | Medial lemniscus | R | 0.1418 |
|  |  | Superior cerebellar peduncle | R | 0.1215 |
|  |  | Superior cerebellar peduncle | L | 0.0608 |
|  |  | Cerebral peduncle | R | 0.3645 |
|  |  | Cerebral peduncle | L | 0.567 |
|  |  | Anterior limb of internal capsule | R | 0.9316 |
|  |  | Anterior limb of internal capsule | L | 0.3645 |
|  |  | Posterior limb of internal capsule | R | 1.4986 |
|  |  | Posterior limb of internal capsule | L | 0.9923 |
|  |  | Retrolenticular part of internal capsule | R | 1.2556 |
|  |  | Retrolenticular part of internal capsule | L | 1.0328 |
|  |  | Anterior corona radiata | R | 1.4986 |
|  |  | Anterior corona radiata | L | 2.3694 |
|  |  | Superior corona radiata | R | 1.5998 |
|  |  | Superior corona radiata | L | 2.7136 |
|  |  | Posterior corona radiata | R | 1.4176 |
|  |  | Posterior corona radiata | L | 1.3973 |
|  |  | Posterior thalamic radiation (include optic radiation) | R | 1.5998 |
|  |  | Posterior thalamic radiation (include optic radiation) | L | 1.9036 |
|  |  | Sagittal stratum (include inferior longitidinal fasciculus and inferior fronto-occipital fasciculus) | R | 0.8505 |
|  |  | Sagittal stratum (include inferior longitidinal fasciculus and inferior fronto-occipital fasciculus) | L | 0.6278 |
|  |  | External capsule | R | 1.3568 |
|  |  | External capsule | L | 1.9644 |
|  |  | Cingulum (cingulate gyrus) | L | 0.2228 |
|  |  | Fornix (cres) / Stria terminalis (can not be resolved with current resolution) | R | 0.4658 |
|  |  | Fornix (cres) / Stria terminalis (can not be resolved with current resolution) | L | 0.729 |
|  |  | Superior longitudinal fasciculus | R | 1.9239 |
|  |  | Superior longitudinal fasciculus | L | 1.3771 |
|  |  | Superior fronto-occipital fasciculus (could be a part of anterior internal capsule) | L | 0.0203 |
|  |  | Uncinate fasciculus | L | 0.162 |
|  |  | Tapetum | R | 0.0608 |
|  |  | Tapetum | L | 0.0203 |
|  | RD: HC < BD_chron_ | Genu of corpus callosum |  | 3.3472 |
|  |  | Body of corpus callosum |  | 7.5765 |
|  |  | Splenium of corpus callosum |  | 2.9061 |
|  |  | Cerebral peduncle | R | 0.0259 |
|  |  | Cerebral peduncle | L | 0.6746 |
|  |  | Anterior limb of internal capsule | R | 1.0119 |
|  |  | Anterior limb of internal capsule | L | 0.3892 |
|  |  | Posterior limb of internal capsule | R | 1.7385 |
|  |  | Posterior limb of internal capsule | L | 1.5568 |
|  |  | Retrolenticular part of internal capsule | R | 1.5568 |
|  |  | Retrolenticular part of internal capsule | L | 1.1936 |
|  |  | Anterior corona radiata | R | 1.5828 |
|  |  | Anterior corona radiata | L | 2.958 |
|  |  | Superior corona radiata | R | 2.0239 |
|  |  | Superior corona radiata | L | 3.7883 |
|  |  | Posterior corona radiata | R | 1.9201 |
|  |  | Posterior corona radiata | L | 1.6347 |
|  |  | Posterior thalamic radiation (include optic radiation) | R | 1.972 |
|  |  | Posterior thalamic radiation (include optic radiation) | L | 2.1536 |
|  |  | Sagittal stratum (include inferior longitidinal fasciculus and inferior fronto-occipital fasciculus) | R | 0.8563 |
|  |  | Sagittal stratum (include inferior longitidinal fasciculus and inferior fronto-occipital fasciculus) | L | 0.7784 |
|  |  | External capsule | R | 0.7784 |
|  |  | External capsule | L | 1.4011 |
|  |  | Cingulum (cingulate gyrus) | L | 0.3373 |
|  |  | Fornix (cres) / Stria terminalis (can not be resolved with current resolution) | R | 0.4411 |
|  |  | Fornix (cres) / Stria terminalis (can not be resolved with current resolution) | L | 0.7784 |
|  |  | Superior longitudinal fasciculus | R | 2.2314 |
|  |  | Superior longitudinal fasciculus | L | 1.7644 |
|  |  | Superior fronto-occipital fasciculus (could be a part of anterior internal capsule) | L | 0.0259 |
|  |  | Uncinate fasciculus | L | 0.2076 |
|  |  | Tapetum | R | 0.0778 |
| Analysis 3: effect of GAF | FA: positive effect | Genu of corpus callosum |  | 3.2348 |
|  |  | Body of corpus callosum |  | 3.6969 |
|  |  | Splenium of corpus callosum |  | 2.1627 |
|  |  | Cerebral peduncle | R | 0.2773 |
|  |  | Cerebral peduncle | L | 0.0739 |
|  |  | Anterior limb of internal capsule | R | 0.7763 |
|  |  | Anterior limb of internal capsule | L | 0.5545 |
|  |  | Posterior limb of internal capsule | R | 1.2015 |
|  |  | Posterior limb of internal capsule | L | 0.9982 |
|  |  | Retrolenticular part of internal capsule | R | 0.9982 |
|  |  | Retrolenticular part of internal capsule | L | 0.9612 |
|  |  | Anterior corona radiata | R | 2.7357 |
|  |  | Anterior corona radiata | L | 2.6433 |
|  |  | Superior corona radiata | R | 1.8669 |
|  |  | Superior corona radiata | L | 1.8854 |
|  |  | Posterior corona radiata | R | 1.5527 |
|  |  | Posterior corona radiata | L | 1.1275 |
|  |  | Posterior thalamic radiation (include optic radiation) | R | 1.719 |
|  |  | Posterior thalamic radiation (include optic radiation) | L | 1.4787 |
|  |  | Sagittal stratum (include inferior longitidinal fasciculus and inferior fronto-occipital fasciculus) | R | 0.7579 |
|  |  | Sagittal stratum (include inferior longitidinal fasciculus and inferior fronto-occipital fasciculus) | L | 0.8872 |
|  |  | External capsule | R | 0.7763 |
|  |  | External capsule | L | 0.8133 |
|  |  | Cingulum (cingulate gyrus) | L | 0.3697 |
|  |  | Fornix (cres) / Stria terminalis (can not be resolved with current resolution) | R | 0.3882 |
|  |  | Fornix (cres) / Stria terminalis (can not be resolved with current resolution) |  | 0.4067 |
|  |  | Superior longitudinal fasciculus | R | 1.793 |
|  |  | Superior longitudinal fasciculus | L | 1.9224 |
|  |  | Superior fronto-occipital fasciculus (could be a part of anterior internal capsule) | R | 0.1848 |
|  |  | Uncinate fasciculus | L | 0.0185 |
|  |  | Tapetum | R | 0.0555 |
|  |  | Tapetum | L | 0.037 |
|  | RD: negative effect | Genu of corpus callosum |  | 3.0616 |
|  |  | Body of corpus callosum |  | 3.5312 |
|  |  | Splenium of corpus callosum |  | 1.7468 |
|  |  | Cerebral peduncle | R | 0.0376 |
|  |  | Cerebral peduncle | L | 0.0188 |
|  |  | Anterior limb of internal capsule | R | 0.3381 |
|  |  | Anterior limb of internal capsule | L | 0.2442 |
|  |  | Posterior limb of internal capsule | R | 0.7513 |
|  |  | Posterior limb of internal capsule | L | 0.5259 |
|  |  | Retrolenticular part of internal capsule | R | 1.0331 |
|  |  | Retrolenticular part of internal capsule | L | 0.8264 |
|  |  | Anterior corona radiata | R | 3.2119 |
|  |  | Anterior corona radiata | L | 3.55 |
|  |  | Superior corona radiata | R | 2.1412 |
|  |  | Superior corona radiata | L | 2.16 |
|  |  | Posterior corona radiata | R | 1.559 |
|  |  | Posterior corona radiata | L | 1.296 |
|  |  | Posterior thalamic radiation (include optic radiation) | R | 1.5214 |
|  |  | Posterior thalamic radiation (include optic radiation) | L | 1.3336 |
|  |  | Sagittal stratum (include inferior longitidinal fasciculus and inferior fronto-occipital fasciculus) | R | 0.7513 |
|  |  | Sagittal stratum (include inferior longitidinal fasciculus and inferior fronto-occipital fasciculus) | L | 0.8828 |
|  |  | External capsule | R | 0.9767 |
|  |  | External capsule | L | 0.3757 |
|  |  | Cingulum (cingulate gyrus) | R | 0.0751 |
|  |  | Cingulum (cingulate gyrus) | L | 0.4132 |
|  |  | Fornix (cres) / Stria terminalis (can not be resolved with current resolution) | R | 0.2066 |
|  |  | Fornix (cres) / Stria terminalis (can not be resolved with current resolution) | L | 0.3757 |
|  |  | Superior longitudinal fasciculus | R | 2.0098 |
|  |  | Superior longitudinal fasciculus | L | 2.4606 |
|  |  | Superior fronto-occipital fasciculus (could be a part of anterior internal capsule) | R | 0.1878 |
|  |  | Superior fronto-occipital fasciculus (could be a part of anterior internal capsule) | L | 0.0376 |
|  |  | Uncinate fasciculus | L | 0.0188 |
|  |  | Tapetum | R | 0.0563 |
|  |  | Tapetum | L | 0.0188 |
|  | MD: negative effect | Genu of corpus callosum |  | 2.4287 |
|  |  | Body of corpus callosum |  | 2.8501 |
|  |  | Splenium of corpus callosum |  | 1.4126 |
|  |  | Anterior limb of internal capsule | R | 0.3717 |
|  |  | Anterior limb of internal capsule | L | 0.0991 |
|  |  | Posterior limb of internal capsule | R | 0.1487 |
|  |  | Posterior limb of internal capsule | L | 0.0248 |
|  |  | Retrolenticular part of internal capsule | R | 1.0409 |
|  |  | Retrolenticular part of internal capsule | L | 0.7931 |
|  |  | Anterior corona radiata | R | 3.6927 |
|  |  | Anterior corona radiata | L | 4.5849 |
|  |  | Superior corona radiata | R | 2.627 |
|  |  | Superior corona radiata | L | 2.627 |
|  |  | Posterior corona radiata | R | 1.7596 |
|  |  | Posterior corona radiata | L | 1.5118 |
|  |  | Posterior thalamic radiation (include optic radiation) | R | 1.6605 |
|  |  | Posterior thalamic radiation (include optic radiation) | L | 1.1896 |
|  |  | Sagittal stratum (include inferior longitidinal fasciculus and inferior fronto-occipital fasciculus) | R | 0.917 |
|  |  | Sagittal stratum (include inferior longitidinal fasciculus and inferior fronto-occipital fasciculus) | L | 1.0409 |
|  |  | External capsule | R | 0.6196 |
|  |  | External capsule | L | 0.0743 |
|  |  | Cingulum (cingulate gyrus) | R | 0.0496 |
|  |  | Cingulum (cingulate gyrus) | L | 0.4957 |
|  |  | Fornix (cres) / Stria terminalis (can not be resolved with current resolution) | R | 0.0248 |
|  |  | Fornix (cres) / Stria terminalis (can not be resolved with current resolution) | L | 0.1487 |
|  |  | Superior longitudinal fasciculus | R | 2.7261 |
|  |  | Superior longitudinal fasciculus | L | 3.3457 |
|  |  | Superior fronto-occipital fasciculus (could be a part of anterior internal capsule) | R | 0.223 |
|  |  | Superior fronto-occipital fasciculus (could be a part of anterior internal capsule) | L | 0.0248 |
|  |  | Uncinate fasciculus | L | 0.0743 |
|  |  | Tapetum | R | 0.0248 |
|  |  | Tapetum | L | 0.0248 |
| Analysis 3: effect of GAF in the whole BD sample (*n*=151) | FA: positive effect | Genu of corpus callosum |  | 7.7892 |
|  |  | Body of corpus callosum |  | 2.291 |
|  |  | Splenium of corpus callosum |  | 2.291 |
|  |  | Cerebral peduncle | R | 0.1145 |
|  |  | Cerebral peduncle | L | 0.3436 |
|  |  | Anterior limb of internal capsule | R | 1.8328 |
|  |  | Anterior limb of internal capsule | L | 3.8946 |
|  |  | Posterior limb of internal capsule | R | 5.0401 |
|  |  | Posterior limb of internal capsule | L | 3.3219 |
|  |  | Retrolenticular part of internal capsule | R | 5.0401 |
|  |  | Retrolenticular part of internal capsule | L | 0.3436 |
|  |  | Anterior corona radiate | R | 4.9255 |
|  |  | Anterior corona radiate | L | 10.6529 |
|  |  | Superior corona radiate | R | 1.6037 |
|  |  | Superior corona radiate | L | 5.1546 |
|  |  | Posterior corona radiate | R | 0.9164 |
|  |  | Posterior thalamic radiation (include optic radiation) | R | 0.3436 |
|  |  | Sagittal stratum (include inferior longitidinal fasciculus and inferior fronto-occipital fasciculus) | R | 1.9473 |
|  |  | Sagittal stratum (include inferior longitidinal fasciculus and inferior fronto-occipital fasciculus) | L | 1.1455 |
|  |  | External capsule | R | 1.7182 |
|  |  | External capsule | L | 1.0309 |
|  |  | Fornix (cres) / Stria terminalis (can not be resolved with current resolution) | R | 1.26 |
|  |  | Fornix (cres) / Stria terminalis (can not be resolved with current resolution) | L | 1.26 |
|  |  | Superior fronto-occipital fasciculus (could be a part of anterior internal capsule) | R | 0.6873 |
| Note: AD=axial diffusivity, BD=participants with bipolar disorder, FA=fractional anisotropy, HC=healthy controls, L=left, k=voxel count, MD=mean diffusivity, R=right, RD=radial diffusivity. | | | | |

1. Hassel S, Almeida JR, Kerr N, Nau S, Ladouceur CD, Fissell K, *et al.* (2008): Elevated striatal and decreased dorsolateral prefrontal cortical activity in response to emotional stimuli in euthymic bipolar disorder: no associations with psychotropic medication load. *Bipolar Disord* 10: 916–927.

2. Vogelbacher C, Möbius TWD, Sommer J, Schuster V, Dannlowski U, Kircher T, *et al.* (2018): The Marburg-Münster Affective Disorders Cohort Study (MACS): A quality assurance protocol for MR neuroimaging data. *NeuroImage* 172: 450–460.

3. Oguz I, Farzinfar M, Matsui J, Budin F, Liu Z, Gerig G, *et al.* (2014): DTIPrep: quality control of diffusion-weighted images. *Front Neuroinform* 8: 4.

4. Jenkinson M, Beckmann CF, Behrens TEJ, Woolrich MW, Smith SM (2012): FSL. *NeuroImage* 62: 782–90.

5. Smith SM, Jenkinson M, Woolrich MW, Beckmann CF, Behrens TEJ, Johansen-Berg H, *et al.* (2004): Advances in functional and structural MR image analysis and implementation as FSL. *NeuroImage* 23: S208-19.

6. Woolrich MW, Jbabdi S, Patenaude B, Chappell M, Makni S, Behrens T, *et al.* (2009): Bayesian analysis of neuroimaging data in FSL. *NeuroImage* 45: S173-86.

7. Andersson JLR, Sotiropoulos SN (2016): An integrated approach to correction for off-resonance effects and subject movement in diffusion MR imaging. *Neuroimage* 125: 1063–1078.

8. Smith SM (2002): Fast robust automated brain extraction. *Human Brain Mapping* 17: 143–155.

9. Behrens TEJ, Woolrich MW, Jenkinson M, Johansen-Berg H, Nunes RG, Clare S, *et al.* (2003): Characterization and propagation of uncertainty in diffusion-weighted MR imaging. *Magn Reson Med* 50: 1077–1088.

10. Alexander AL, Hurley SA, Samsonov AA, Adluru N, Hosseinbor AP, Mossahebi P, *et al.* (2011): Characterization of cerebral white matter properties using quantitative magnetic resonance imaging stains. *Brain Connect* 1: 423–446.

11. Jones DK, Knösche TR, Turner R (2013): White matter integrity, fiber count, and other fallacies: the do’s and don’ts of diffusion MRI. *Neuroimage* 73: 239–254.

12. Feldman HM, Yeatman JD, Lee ES, Barde LHF, Gaman-Bean S (2010): Diffusion tensor imaging: A review for pediatric researchers and clinicians. *Journal of Developmental and Behavioral Pediatrics* 31: 346–356.

13. Smith SM, Jenkinson M, Johansen-Berg H, Rueckert D, Nichols TE, Mackay CE, *et al.* (2006): Tract-based spatial statistics: voxelwise analysis of multi-subject diffusion data. *Neuroimage* 31: 1487–1505.

14. Engvig A, Fjell AM, Westlye LT, Moberget T, Sundseth Ø, Larsen VA, Walhovd KB (2012): Memory training impacts short-term changes in aging white matter: a longitudinal diffusion tensor imaging study. *Hum Brain Mapp* 33: 2390–2406.

15. Leenders AEM, Damatac CG, Soheili-Nezhad S, Chauvin RJM, Mennes MJJ, Zwiers MP, *et al.* (2021): Associations between attention-deficit hyperactivity disorder (ADHD) symptom remission and white matter microstructure: A longitudinal analysis. *JCPP Adv* 1: e12040.

16. Madhyastha T, Mérillat S, Hirsiger S, Bezzola L, Liem F, Grabowski T, Jäncke L (2014): Longitudinal reliability of tract-based spatial statistics in diffusion tensor imaging. *Hum Brain Mapp* 35: 4544–4555.

17. Jenkinson M, Smith S (2001): A global optimisation method for robust affine registration of brain images. *Medical Image Analysis* 5: 143–156.

18. Smith SM, De Stefano N, Jenkinson M, Matthews PM (2001): Normalized accurate measurement of longitudinal brain change. *J Comput Assist Tomogr* 25: 466–475.

19. Oishi K, Faria A, Jiang H, Li X, Akhter K, Zhang J, *et al.* (2009): Atlas-based whole brain white matter analysis using large deformation diffeomorphic metric mapping: Application to normal elderly and Alzheimer’s disease participants. *NeuroImage* 46: 486–499.

20. Borenstein M (2009): Effect sizes for continuous data. *The Handbook of Research Synthesis and Meta-Analysis, 2nd Ed*. New York, NY, US: Russell Sage Foundation, pp 221–235.
